# Surface glia for modeling ALS-FTD-associated mutant C9orf72 toxicity in the nervous system of *Drosophila*

**DOI:** 10.1016/j.gendis.2025.101629

**Published:** 2025-04-05

**Authors:** Yanan Wei, Brittany Anne Snow, Ciara Crowley Stevenson, Hongyu Miao, Jasdeep Kaur, Seung Gee Lee, Nam Chul Kim, Woo Jae Kim

**Affiliations:** a The HIT Center for Life Sciences, Harbin Institute of Technology, Harbin, Heilongjiang 150080, China; b Department of Cellular and Molecular Medicine, University of Ottawa, Ottawa K1H 8M5, Canada; c Department of Pharmacy Practice and Pharmaceutical Sciences, College of Pharmacy, University of Minnesota, Duluth, MN 55812, USA; d Medical and Health Research Institute, Zhengzhou Research Institute of HIT, Zhengzhou, Henan 450044, China

The GGGGCC repeat expansion in the chromosome 9 open reading frame 72 gene (*C9orf72*) is a leading genetic cause of amyotrophic lateral sclerosis and frontotemporal dementia (ALS-FTD). Despite the prevalence of this mutation, effective therapies remain elusive due to the complexity of the disease. Our study leverages *Drosophila* models to investigate the role of surface glia in mediating the toxicity associated with mutant C9orf72. With widely used neuronal and glial GAL4 (galectin 4) drivers and recently developed GAL4 drivers that separately mark each subtype of the glial system in fruit flies, we analyzed the toxicity of various *C9orf72* mutants. Our findings demonstrate that surface glia, a model for the blood–brain barrier in vertebrates, exhibit heightened vulnerability to the expression of dipeptide repeat (DPR) originating from the mutant *C9orf72* gene. This susceptibility results in pronounced developmental toxicity, as well as deficits in adult motor function and reduced lifespan. Significantly, the expression of GR100 DPR in surface glia did not lead to massive cell death of neurons or glia in the central nervous system. Additionally, the drug ursodeoxycholic acid (UDCA), which is intended to rescue the pan-neuronal *Drosophila* model, tended to negatively impact the locomotor activity and lifespan of glial cell models. Our findings suggest that glial cells may play a more substantial role in ALS-FTD pathogenesis than previously recognized, offering new avenues for therapeutic intervention.

We hypothesized that specific glial subtypes in *Drosophila* may be particularly vulnerable to the expression of C9orf72 DPR, leading to distinct pathological phenotypes.^1^ To test this hypothesis, we employed a series of GAL4 drivers to target six different glial subtypes in *Drosophila*, allowing for the tissue-specific expression of various C9orf72-related constructs.^2^ We analyzed the effects of these expressions on lethality, locomotor activity, and lifespan, providing insights into the cellular mechanisms underlying ALS-FTD pathology as previously described.^3^

We first assessed the developmental toxicity of C9orf72 DPR expression in glial cells by measuring the egg-to-adult viability. Flies expressing *UAS-C9orf72* variants under the control of glial subtype-specific GAL4 drivers were subjected to this analysis. The neuronal and glial expression of C9orf72 DPR variants exerted equivalent developmental toxicity (Data S1). Interestingly, the expression of GR100 DPR in perineurial glia (PNG) and subperineurial glia (SPG) using their respective GAL4 drivers resulted in a significant reduction in the number of eclosed adults, indicating high levels of lethality (Fig. 1A; Data S2). This effect was not observed with other glial subtypes, suggesting that PNG and SPG are particularly sensitive to the toxic effects of GR100 DPR. We also observed that GR100 may affect specific developmental steps or signaling pathways during the development of SPG and PNG, including some male-specific pathways (Data S3).

**Figure 1:**
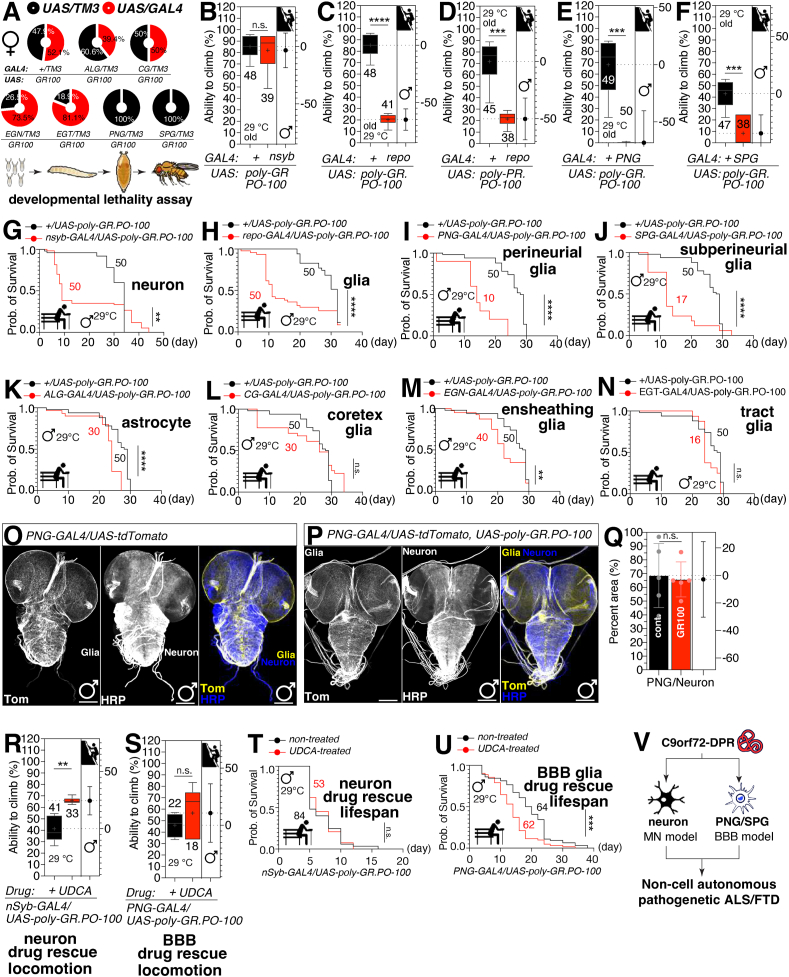
Lethality, lifespan, climbing ability, and rescue assays of flies expressing GR100 DPR. **(A)** The percentage of female flies eclosed from tissue-specific expression of *UAS-GR100* crossed with control (+) and each subtype glia-*GAL4* drivers (ALG, CG, EGN, EGT, PNG, and SPG). **(B)** Climbing test of 20-day-old male flies expressing *C9orf72* variant GR100 by *nSyb-GAL4* driver. **(C)** Climbing test of 20-day-old male flies expressing *C9orf72* variant GR100 by *repo-GAL4* driver. **(D)** Climbing test of 20-day-old male flies expressing *C9orf72* variant PR100 by *repo-GAL4* driver. **(E, F)** Climbing test of 20-day-old male flies expressing *C9orf72* variant GR100 by *PNG-* (E) and *SPG-GAL4* driver (F). **(G**–**N)** Lifespan assay of male flies expressing GR100 by pan-neuronal or glial drivers and subtype glia*-GAL4* drivers, with *nSyb*-*GAL4* (G), *repo*-*GAL4* (H), *PNG*-*GAL4* (I), *SPG*-*GAL4* (J), *ALG*-*GAL4* (K), *CG*-*GAL4* (L), *EGN*-*GAL4* (M), and *EGT*-*GAL4* (N). **(O, P)** Larval central nervous system immunostaining and quantification with GR100 expression in surface glia. The third instar larval central nervous system expressing *UAS-tdTomato* with *PNG-GAL4* only (O) or with *UAS-GR100* (P) were immunostained with anti-DsRed (yellow) and anti-HRP (blue, neuronal staining) antibodies. Scale bars represent 100 μm. **(Q)** Percent area quantified from the ratio of glia/neuron. **(R, S)** Climbing test of non-treated or UDCA-treated flies expressing GR100 variant by *nSyb*- (R) and *PNG-GAL4* (S) drivers. **(T, U)** Lifespan assay of non-treated or UDCA-treated flies expressing GR100 variant by *nSyb*- (T) and *PNG-GAL4* (U) drivers. **(V)** Schematic diagram and summary of this manuscript. DPR, dipeptide repeat; GAL4, galectin 4; CG, cortex glia; ALG, astrocyte-like glia; EGT, tract ensheathing glia; EGN, ensheathing glia; SPG, subperineurial glia; PNG, perineurial glia; UDCA, ursodeoxycholic acid.

Although robust developmental phenotypes are valuable for pinpointing pathways and cellular processes affected by mutant *C9orf72*, examining adult-onset abnormalities may yield more pertinent insights into ALS and FTD pathogenesis, given these diseases' typical late onset. To test the toxicity of *C9orf72* in adult animals, we expressed *UAS-C9orf72* variants using *tub-GAL80*^*ts*^ and temperature shift for adult-specific expression of DPR and then examined their climbing ability and life span.

When we expressed various mutant *C9orf72* constructs pan-neuronally using *nSyb-GAL4* and *tub-GAL80*^*ts*^, we could detect the declined climbing ability only in old males (Fig. 1B; Data S4, 5). Interestingly, only males expressing DPR in glia showed severe locomotion defects (Fig. 1C, D; Data S6, 7). The observation that certain C9orf72 DPR (G4C2, GR100, and PR100) primarily affect climbing ability in male flies suggests that sex-specific factors may influence the susceptibility and/or severity of DPR-induced neurotoxicity. Male and female flies may exhibit sex-specific differences in neuronal circuitry, glial cells, and motor function, which could influence their susceptibility to the effects of DPR. Therefore, our findings suggest that glial expression of *C9orf72* DPR could produce locomotor defects that may be useful to elucidate non-cell autonomous mechanisms of ALS and FTD. The flies expressing GR100 DPR in PNG and SPG exhibited a marked decline in climbing ability, especially in older flies (Fig. 1E, F). This decline was not observed in flies expressing other DPR variants or in control groups (Data S8), highlighting the specificity of the locomotor deficits associated with GR100 DPR expression in surface glia.

Next, we examined the influence of mutant *C9orf72* variants' expression on lifespan. Our data showed that the expression of mutant *C9orf72* variants in all neurons or all glia had a comparable but slightly distinct influence on adult lifespan, which indicates the possibility of application as a non-cell autonomous model (Fig. 1G, H; Data S9, 10). In aggregate, the mean lifespan of male subjects was considerably extended relative to their female counterparts (Data S11A). To further determine which subtype of glia was responsible for mutant *C9orf72*'s influence on lifespan, GR100 was expressed in six distinct subtypes of glia with subtype-specific *GAL4* drivers. Both male and female fly lifespans were reduced, especially with ALG-, PNG-, and SPG-*GAL4*, while in other subtypes of glial cells, lifespan was not or only mildly affected (Fig. 1I–N; Data S12). These findings demonstrate that surface glia is the primary location at which the *C9orf72* DPR causes lifespan reduction in the adult fruit fly model.

To investigate the mechanisms of glia-mediated *C9orf72* toxicity on developmental lethality, we monitored the neuron and glial cells when we expressed C9orf72 DPR. When GR100 was expressed in SPG or PNG, no significant cell death or glial mass reduction was observed in larval brains (Fig. 1O–Q; Data S13), although it resulted in 100% lethality (Fig. 1A; Data S1–3). The quantitative reverse transcription PCR analysis of GAL4 mRNA expression levels in different glial subtypes revealed no significant differences between control and experimental groups, suggesting that the observed toxicity was not primarily due to variations in GAL4 driver expression levels (Data S11B). Interestingly, GR100 expression in ALG was associated with a modest decrease in neuronal mass (Data S14). This finding suggests that the toxic effects of GR100 DPR on surface glia may not be mediated by direct cell death.

To investigate whether the glial cell model acted differently from the neuronal cell model, we used a US FDA-approved drug, UDCA,^4^ which has been demonstrated to have neuroprotective effects in FTD, suggesting potential therapeutic benefits in neurodegenerative diseases.^5^ UDCA treatment improved the climbing ability of young flies expressing GR100 DPR in neurons but harmed the climbing ability of flies expressing GR100 DPR in surface glia (Fig. 1R, S; Data S15). Similarly, our data demonstrates that while UDCA treatment did not affect the lifespan of flies expressing GR100 DPR in neurons, it tended to reduce the lifespan of flies expressing GR100 DPR in surface glia (Fig. 1T, U; Data S16). These findings suggest that the glial cell models may have distinct pathogenic mechanisms compared with neuronal models and could be valuable for investigating disease mechanisms and evaluating therapeutic interventions.

In conclusion, our results demonstrate that surface glia, particularly PNG and SPG, are highly susceptible to the toxic effects of mutant *C9orf72*, leading to significant developmental toxicity, impaired locomotor function, and reduced lifespan in *Drosophila*. The distinct response to UDCA treatment in glial versus neuronal models underscores the complexity of ALS-FTD pathogenesis and highlights the importance of considering non-neuronal cell types in disease research and therapeutic development. Our findings provide valuable insights into the cellular mechanisms underlying ALS-FTD and offer new avenues for investigating disease mechanisms and evaluating therapeutic interventions (Fig. 1V).

Our findings also have broader implications for the use of *Drosophila* models in neurodegenerative disease research. By revealing the vulnerability of specific glial subtypes to C9orf72 DPR, our study expands the utility of *Drosophila* as a model for investigating the cellular and molecular mechanisms underlying ALS-FTD. The distinct phenotypes observed in our glial models provide a unique opportunity for high-throughput screening of potential therapeutics and for investigating the genetic modifiers that influence disease progression.

One limitation of this study stems from the absence of a *C9orf72* homolog in *Drosophila*. Therefore, we can only use an overexpression model in fruit flies and cannot model the physiologically normal levels of mutant *C9orf72* expression found in patients. Additionally, the use of multiple independent drivers to assess toxicity in each tissue or cell makes it difficult to determine or compare how much DPR is expressed to cause toxicity in that tissue or cell. However, after testing a number of relatively well-expressed drivers, we show that surface glia are the most vulnerable cells to mutant *C9orf72* in fruit flies. Using these cells, we can identify which intracellular signaling or important cellular processes are affected by the *C9orf72* mutation at the organismal level during development or in the adult state. As we demonstrated with UDCA, this will lead us to find a way to ameliorate *C9orf72* toxicity derived from non-neuronal mechanisms, which can result in loss of motor skills or early death in fruit flies.

## CRediT authorship contribution statement

**Yanan Wei:** Writing – review & editing, Visualization, Methodology, Data curation, Conceptualization. **Brittany Anne Snow:** Visualization, Methodology, Data curation. **Ciara Crowley Stevenson:** Visualization, Methodology, Data curation. **Hongyu Miao:** Writing – review & editing. **Jasdeep Kaur:** Visualization, Methodology, Data curation. **Seung Gee Lee:** Visualization, Methodology, Data curation. **Nam Chul Kim:** Writing – original draft, Supervision, Project administration, Formal analysis, Conceptualization. **Woo Jae Kim:** Writing – review & editing, Writing – original draft, Visualization, Validation, Supervision, Resources, Project administration, Methodology, Investigation, Funding acquisition, Formal analysis, Data curation, Conceptualization.

## Funding

This work was supported by the University of Ottawa Startup grant to W.J.K., University of Ottawa Brain and Mind Research Institute/Center for Neural Dynamics Open Call Project grant to W.J.K., University of Ottawa Interdisciplinary Research Group Funding Opportunity (IRGFO stream 1 and 2) grant to W.J.K., Mitacs Globalink Research Internship Program grant to W.J.K., and startup funds from HIT Center for Life Science to W.J.K. This work was also supported by a Brain Pool Program by the National Research Foundation in Korea to W.J.K., Burroughs Wellcome Fund Collaborative Research Travel grant 1017486 to W.J.K., and NVIDIA Academic Hardware Grant Program to W.J.K.

## Conflict of interests

The authors declared no competing interests.
