# Supplementary material for: Surface glia for modeling ALS-FTD-associated mutant C9orf72 toxicity in the nervous system of Drosophila
Source: Genes Dis. 2025 Apr 5;13(2):101629. doi: 10.1016/j.gendis.2025.101629 (PMC12596593; doi:10.1016/j.gendis.2025.101629)
Supplement: Multimedia component 1 [file mmc1.docx]

**METHODS**

**Fly Rearing and Strains**

*Drosophila melanogaster* was raised on cornmeal-yeast medium at similar densities to yield adults with similar body sizes. Flies were kept in 12 h light: 12 h dark cycles (LD) at 25°C (zeitgeber time (ZT) 0 is the beginning of the light phase, zeitgeber time (ZT) 12 beginning of the dark phase) except for some experimental manipulation (experiments with the flies carrying *tub-GAL80^ts^*). Wild-type flies were Canton-S (CS). To reduce the variation from genetic background, all flies were backcrossed for at least 3 generations to CS strain. All mutants and transgenic lines used here have been described previously.

The following lines were obtained from Bloomington Stock Center (#stock number): *nSyb-GAL4* (#51941), *repo-GAL4* (#7415), *nSyb-GAL4* (#51941), *ALG-GAL4* (#45914), *CG-GAL4* (#39944), *EGN-GAL4* (#39157), *EGT-GAL4* (#39908), *PNG-GAL4* (#40436), *SPG-GAL4* (#50472), *OK6-GAL4* (#64199), *D42-GAL4* (#8816), *UAS-288RO* (#58691), *UAS-G4C2(36)* (#58688), *UAS-GR100* (#58696), *UAS-GA100* (#58697), *UAS-PR100* (#58694), *UAS-PA100* (#58699), *tub-GAL80^ts^* (#7017), *UAS-tdTomato* (#32221), *UAS-mCD8GFP* (#5130), *UAS-RedStinger* (#8547), *UAS-mCD8RFP, lexAop-mCD8GFP* (#32229).

**Lethality Assay**

We evaluated the lethality of *C9orf72* variants expressed by tissue-specific *GAL4*s using two separate methods. First, the effects of tissue-specific *C9orf72* variants expression were evaluated based on the number of eggs deposited and the percentage of eggs that eclosed throughout development. The number of eggs generated by a particular cross was measured twenty-four hours after the cross was done. The adults were transferred to a second vial to generate a replica, which was then destroyed after two days. Eggs were counted in the vial using visual discrimination under the microscope. The percentage of egg to adult viability was determined by dividing the number of flies of each cross that eclosed by the number of eggs deposited, then multiplying by 100. Male and female offspring were counted separately. Second, to measure the effects of each cross for lethality, we used *TM3* balancer chromosome. Virgin female *UAS-GR100* flies and male *GAL4/TM3* flies were crossed and the proportion of progeny with the *TM3* balancer was measured compared the progeny without *TM3*, which have a *GAL4* driver for a specific glial cell type. For control experiments, we used male *TM3, y[+] Ser[1]/Sb[1]* then measures the ratio of *Ser[1]* and *Sb[1]* marker phenotype.

**Climbing Assay**

For climbing assay, we modified the conventional RING assay ^1^. In brief, 40-50 aged flies were placed in an empty vial and were tapped to the bottom of the tube. We used 5 days old adults as young flies and 20 days old as old files. After tapping of flies, we recorded 10 seconds of video clip. This experiment was done five times with 5-minute intervals. With recorded video files, we captured the position of flies 10 seconds after tapping the vial. This captured image file was then loaded in ImageJ to perform particle analysis. For quantifying the location of flies inside a vial, we used the “analyze particles” function of ImageJ ^2^. The position of pixels was normalized by height of vial then only the particles above the midline (4 cm) of vial were counted.

**Lifespan Assay and Statistical Analysis**

For lifespan analysis, we used conventional procedure as previously described ^3^. Briefly, 50 flies were aged by sex before being raised in typical 12 h light: 12 h dark cycles at either 29°C or 20°C for each experimental objective. The number of dead flies was recorded daily for forty days. Every three to four days, the surviving flies were transferred to fresh vials. Some of the lifespan data indicating early mortality may have been influenced by the high temperature of 29°C, which was intended to induce disease gene expression. To compare the survival curves of each genotype, the data was analyzed by Gehan-Breslow-Wilcoxon test, performed by GraphPad (Prism). **** = p<0.0001, *** = p < 0.001, ** = p < 0.01, * = p < 0.05, n.s. stands for non-significant differences.

**Drug treatment and analysis**

The potential ALS drug, ursodeoxycholic acid (UDCA, SIGMA Lot # SLCD5862), was used to rescue the abnormal phenotypes of flies expressing disease genes in glial cells and neuronal cells. The concentration was decided according to a previous ^43^. The drug was dissolved into ethanol first to help better dispense into normal food at a concentration of 120 µM with the final concentration of ethanol around 9%, while control groups were treated with normal food only. Flies with different phenotypes were reared in vials containing drug food and climbing and lifespan assays were conducted to check whether the drug can improve glia and neuron phenotypes.

**Larval CNS Dissection and Immunostaining**

As described before ^5^, wandering third instar larval CNS was dissected then fixed in 4% formaldehyde for 30 min at room temperature, washed with 1% PBT three times (30 min each) and blocked in 5% normal donkey serum for 30 min. The brains were then incubated with primary antibodies in 1% PBT at 4°C overnight followed with fluorophore-conjugated secondary antibodies for 1 hour at room temperature. Brains were mounted with anti-fade mounting solution (Invitrogen, catalog #S2828) on slides for imaging. Primary antibodies: rabbit anti-DsRed express (Clontech, 1:250) and goat anti-HRP (Jackson Lab, 1:500). Fluorophore-conjugated secondary antibodies: RRX-conjugated donkey anti-rabbit (Jackson Lab, 1:100) and Dylight 649-conjugated donkey anti-goat (Jackson Lab, 1:100). Confocal images of the larval brain were scanned from the ventral side upward and the dorsal side downward.

**Adult Leg Dissection and Live Imaging**

Aged flies were dissected under chilled PBS buffer then washed several times with cold PBS. Washed legs and wings then transferred to slide glass then mounted with cover glass filled with 50% glycerol solution. Dissected samples were immediately imaged by Zeiss AxioImager M2. DIC channel was used for taking images of external organs.

**Quantitative Analysis of tdTomato Fluorescence**

To quantify the tdTomoto signals in larval CNS, we measured fluorescence intensity using the measure tool of ImageJ (National Institutes of Health, http://rsb.info.nih.gov/ij) as described previously ^6,7^. Fluorescence was quantified in a manually set region of interest (ROI). Percent of area represents percent of area that is covered with particles normalized by total area. Mean intensity represents the mean fluorescence intensity of tdTomato signal thus can be interpreted as the expression level. Prior to quantification, we removed any broken cell debris from outside the larval brain region using ImageJ function to ensure accurate measurement of neuronal and glial mass. Previous studies have shown that HRP can serve as a reliable marker for assessing neuronal populations, but it is crucial to interpret these results within the context of the specific experimental conditions and the biological significance of the observed differences (Li 2010,Meloni 2020).

**Quantitative RT-PCR**

The expression levels of *GAL4* in flies with different subtype glias were analyzed by quantitative real-time RT-PCR with SYBR Green qPCR MasterMix kit (Selleckchem) and *tub-GAL4* as control. The primer of RT-PCR is *GAL4*, F:5’- GCAACTATCGCCCAATACC-3’; R: 5’- GCCACCAAACAAAGCAGAC-3’. qPCR reactions were performed in triplicate, and the specificity of each reaction was evaluated by dissociation curve analysis. Each experiment was replicated three times. PCR results were recorded as threshold cycle numbers (Ct). The fold change in the target gene expression, normalized to the expression of internal control gene (GAPDH) and relative to the expression at time point 0, was calculated using the 2 ^−ΔΔCT^ method as previously described^8^. The results are presented as the mean ± SD of three independent experiments.

**Statistical Analysis**

Statistical analysis of climbing assay was similar with our previous studies ^5,7,9^. 40-50 adults were used for climbing assay. Statistical comparisons were made between control groups and experimental groups within each experiment. As climbing data of adults showed normal distribution (Kolmogorov-Smirnov tests, p > 0.05), we used two-sided Student's t tests. Each figure shows the mean ± standard error (s.e.m) (**** = p<0.0001, *** = p < 0.001, ** = p < 0.01, * = p < 0.05). All analysis was done in GraphPad (Prism). Individual tests and significance are detailed in figure legends. Besides traditional t-test for statistical analysis, we added estimation statistics for all climbing assays and two group comparing graphs. In short, ‘estimation statistics’ is a simple framework that—while avoiding the pitfalls of significance testing—uses familiar statistical concepts: means, mean differences, and error bars. More importantly, it focuses on the effect size of one's experiment/intervention, as opposed to significance testing ^10^. In comparison to typical Null Hypothesis Significance Testing (NHST) plots, estimation graphics have the following five significant advantages such as (1) avoid false dichotomy, (2) display all observed values (3) visualize estimate precision (4) show mean difference distribution. And most importantly (5) by focusing attention on an effect size, the difference diagram encourages quantitative reasoning about the system under study ^11^. Thus, we conducted a reanalysis of all of our two group data sets using both standard t-tests and estimate statistics. In 2019, the Society for Neuroscience journal eNeuro instituted a policy recommending the use of estimation graphics as the preferred method for data presentation ^12^

**REFERENCES**

1. Gargano JW, Martin I, Bhandari P, Grotewiel MS. Rapid iterative negative geotaxis (RING): a new method for assessing age-related locomotor decline in Drosophila. *Exp Gerontol*. 2005;40(5):386-395. doi:10.1016/j.exger.2005.02.005

2. Grishagin IV. Automatic cell counting with ImageJ. *Anal Biochem*. 2015;473:63-65. doi:10.1016/j.ab.2014.12.007

3. Piper MDW, Partridge L. Protocols to Study Aging in Drosophila. In: Springer New York; 2016:291-302. doi:10.1007/978-1-4939-6371-3_18

4. West RJH, Ugbode C, Fort-Aznar L, Sweeney ST. Neuroprotective activity of ursodeoxycholic acid in CHMP2B Intron5 models of frontotemporal dementia. *Neurobiol Dis*. 2020;144:105047. doi:10.1016/j.nbd.2020.105047

5. Kim WJ, Jan LY, Jan YN. A PDF/NPF Neuropeptide Signaling Circuitry of Male Drosophila melanogaster Controls Rival-Induced Prolonged Mating. *Neuron*. 2013;80(5):1190-1205. doi:10.1016/j.neuron.2013.09.034

6. Kim WJ, Jan LY, Jan YN. A PDF/NPF Neuropeptide Signaling Circuitry of Male Drosophila melanogaster Controls Rival-Induced Prolonged Mating. *Neuron*. 2013;80(5):1190-1205. doi:10.1016/j.neuron.2013.09.034

7. Kim WJ, Lee SG, Auge AC, Jan LY, Jan YN. Sexually satiated male uses gustatory-to-neuropeptide integrative circuits to reduce time investment for mating. *Biorxiv*. Published online 2016:088724. doi:10.1101/088724

8. Livak KJ, Schmittgen TD. Analysis of Relative Gene Expression Data Using Real-Time Quantitative PCR and the 2−ΔΔC T Method. *Methods*. 2001;25(4):402-408. doi:10.1006/meth.2001.1262

9. Kim WJ, Jan LY, Jan YN. Contribution of visual and circadian neural circuits to memory for prolonged mating induced by rivals. *Nat Neurosci*. 2012;15(6):876-883. doi:10.1038/nn.3104

10. Claridge-Chang A, Assam PN. Estimation statistics should replace significance testing. *Nat Methods*. 2016;13(2):108-109. doi:10.1038/nmeth.3729

11. Ho J, Tumkaya T, Aryal S, Choi H, Claridge-Chang A. Moving beyond P values: data analysis with estimation graphics. *Nat Methods*. 2019;16(7):565-566. doi:10.1038/s41592-019-0470-3

12. Bernard C. Estimation Statistics, One Year Later. *Eneuro*. 2021;8(2):ENEURO.0091-21.2021. doi:10.1523/eneuro.0091-21.2021

**FIGURE LEGENDS**

**Fig. SD1.** *Toxicity of mutant C9orf72 variants* in neuron and glial cell populations measured by the percentage of egg to adult lethality. (A-D) The percentage of flies that eclosed from tissue-specific expression of *UAS-288RO, UAS-G4C2(36), UAS-GR100, UAS-GA100, UAS-PR100* and *UAS-PA100* by (A) *nSyb-GAL4* (pan-neuronal driver), (B) *repo-GAL4* (pan-glial driver), (C) *OK6-GAL4* (motor neuron driver), and (D) *D42-GAL4* (motor neuron and peripheral nervous system). See **METHODS** for detailed statistical analysis used in this study.

**Fig. SD2.** The percentage of male flies eclosed from tissue specific expression of *UAS-GR100* crossed with control (+) and each subtype glia-*GAL4* drivers (ALG, CG, EGN, EGT, PNG, and SPG).

**Fig. SD3.** The inducible expression of *GR100* DPR using *tub-GAL80^ts^* at different time points during development. (A) Schematic diagram of GR100 expression by temperature shift from 20°C to 29°C at different time points. (B-C) The percentage of eclosed flies (top) or male/female ratio of eclosed flies (bottom) from tissue-specific expression of *UAS-GR100* by (B) *SPG-GAL4* and (C) *PNG-GAL4*.

**Fig. SD4.** Climbing test of flies expressing *C9orf72* variants by *nSyb-GAL4* driver. (A-D) 288RO expressing flies, 5-day-old females (A) and males (B), 20-day-old females (C) and males (D). (E-H) G4C2(36) expressing flies, 5-day-old females (E) and males (F), 20-day-old females (G) and males (H). (I-L) PR100 expressing flies, 5-day-old females (I) and males (J), 20-day-old females (K) and males (L). (M-P) GR100 expressing flies, 5-day-old females (M) and males (N), 20-day-old females (O) and males (P). Box-and-whisker plot represent the percent of flies crossed midline. All experiments were performed five times after sufficient recovering period. Genotypes are labeled below the graph. Rearing temperature, age, and sex of animals are labeled within the graph. Box represents min to max that show all points of data. The median value and standard error are labeled within the box-and-whisker plot (black lines). Mean value is labeled as cross mark (**+**) within box. Right Y axis represents estimation plot and the black whiskers span the 95% CIs ^11^. Asterisks represent significant differences revealed by unpaired Student's *t* test (* *p<0.05*, ** *p<0.01*, *** *p<0.001,* **** *p<0.0001*). n.s. represent non-significant differences revealed by unpaired Student’s *t* test. The same notations of climbing assay for statistical analysis are used in other figures. See **METHODS** and previous report ^6,7^ for detailed quantification methods.

**Fig. SD5.** Climbing test of flies carrying *C9orf72* variants with *nSyb-GAL4* driver at 20°C. (A-D) 288RO expressing flies, 5-day-old females (A) and males (B), 20-day-old females (C) and males (D). (E-H) G4C2(36) expressing flies, 5-day-old females (E) and males (F), 20-day-old females (G) and males (H). (I-L) PR100 expressing flies, 5-day-old females (I) and males (J), 20-day-old females (K) and males (L). (M-P) PA100 expressing flies, 5-day-old females (M) and males (N), 20-day-old females (O) and males (P). Box-and-whisker plot represent the percent of flies crossed midline. All experiments were performed five times after sufficient recovering period. Genotypes are labeled below the graph. Rearing temperature, age, and sex of animals are labeled within the graph.

**Fig. SD6.** Climbing test of flies expressing *C9orf72* variants by *repo-GAL4* driver. (A-D) 288RO expressing flies, 5-day-old females (A) and males (B), 20-day-old females (C) and males (D). (E-H) G4C2(36) expressing flies, 5-day-old females (E) and males (F), 20-day-old females (G) and males (H). (I-L) PR100 expressing flies, 5-day-old females (I) and males (J), 20-day-old females (K) and males (L). (M-P) GR100 expressing flies, 5-day-old females (M) and males (N), 20-day-old females (O) and males (P). Box-and-whisker plot represent the percent of flies crossed midline. All experiments were performed five times after sufficient recovering period. Genotypes are labeled below the graph. Rearing temperature, age, and sex of animals are labeled within the graph.

**Fig. SD7.** Climbing test of flies carrying *C9orf72* variants with *repo-GAL4* driver at 20°C. (A-D) 288RO expressing flies, 5-day-old females (A) and males (B), 20-day-old females (C) and males (D). (E-H) G4C2(36) expressing flies, 5-day-old females (E) and males (F), 20-day-old females (G) and males (H). (I-L) PR100 expressing flies, 5-day-old females (I) and males (J), 20-day-old females (K) and males (L). (M-P) PA100 expressing flies, 5-day-old females (M) and males (N), 20-day-old females (O) and males (P). Box-and-whisker plot represent the percent of flies crossed midline. All experiments were performed five times after sufficient recovering period. Genotypes are labeled below the graph. Rearing temperature, age, and sex of animals are labeled within the graph.

**Fig. SD8.** Climbing test of flies expressing *C9orf72* variants by PNG and SPG*-GAL4* driver. (A-B) GR100 expressing by PNG-*GAL4* driver flies, 20-day-old females (A) and males (B), (C-D) GR100 expressing by SPG-*GAL4* driver flies, 20-day-old females (C) and males (D). Box-and-whisker plot represent the percent of flies crossed midline. All experiments were performed five times after sufficient recovering period. Genotypes are labeled below the graph. Rearing temperature, age, and sex of animals are labeled within the graph.

**Fig. SD9.** Lifespan assay of flies expressing *C9orf72* variants by *nSyb-GAL4* driver. (A-B) 288RO expressing flies, females (p value=0.9176, median survival=28 for control, 31 for C9 flies) (A) and males (p value=0.0802, median survival=38 for both conditions) (B), (C-D) G4C2(36) expressing flies, females (p value<0.0001, median survival=42 for control, 17 for C9 flies) (C) and males (p value<0.0001, median survival=38 for control, 21 for C9 flies) (D), (E-F) GR100 expressing flies, females (p value=0.0088, median survival=23 for control, 7 for C9 flies) (E) and males (p value=0.0039, median survival=34 for control, 9 for C9 flies) (F) (E-F) PR100 expressing flies, females (p value<0.0001, median survival=20 for control, 13 for C9 flies) (E) and males (p value<0.0001, median survival=34 for control, 13 for C9 flies) (F), (G-H) PA100 expressing flies, females (p value=0.0109, median survival=35 for control, 38 for C9 flies) (G) and males (p value=0.1713, median survival=35 for control, 38 for C9 flies) (H). Each colored dot represents the percentage of survived flies at that day. Colored slope represents the line by the linear regression analysis. The numbers showed in the figures represent sample size of each condition. The survival curves were tested by Gehan-Breslow-Wilcoxon test. See **METHODS** for detailed description of lifespan assay. Genotypes are labeled above the graph by indicated color. Rearing temperature, age, and sex of animals are labeled within the graph. The same notations of lifespan assay for statistical analysis are used in other figures.

**Fig. SD10.** Lifespan assay of flies expressing *C9orf72* variants by *repo-GAL4* driver. (A-B) 288R expressing flies, females (p value=0.0062, median survival=29.5 for control, 25 for C9 flies) (A) and males (p value<0.0001, median survival=30 for control, 25 for C9 flies) (B), (C-D) G4C2(36) expressing flies, females (p value<0.0001, median survival=29 for control, 14.5 for C9 flies) (C) and males (p value<0.0001, median survival=26 for control, 10.5 for C9 flies) (D), (E-F) GR100 expressing flies, females (p value<0.0001, median survival=32 for control, 21 for C9 flies) (E) and males (p value<0.0001, median survival=30 for control, 11 for C9 flies) (F), (G-H) PR100 expressing flies, females (p value=0.8941, median survival=19 for control, 18 for C9 flies) (G) and males (p value<0.0001, median survival=20 for control, 15 for C9 flies) (H), (I-J) PA100 expressing flies, females (p value=0.2967, median survival=20 for control, 16.5 for C9 flies) (I) and males (p value=0.7504, median survival=16.5 for control, 16 for C9 flies) (J). Genotypes are labeled above the graph by indicated color. Rearing temperature, age, and sex of animals are labeled within the graph.

**Fig. SD11.** Control experiments for lifespan assay. (A) Lifespan assay of Canton-S males and females. (p value=0.0002, median survival=24 for males, 31 for female flies) (B) Expression level of *GAL4* mRNA by qRT-PCR.

**Fig. SD12.** Lifespan assay of flies expressing GR100 by subtype glia*-GAL4* drivers. (A-B) with ALG-*GAL4*, females (p value<0.0001, median survival=31 for control, 27 for C9 flies) (A) and males (p value<0.0001, median survival=28.5 for control, 24 for C9 flies) (B), (C-D) with CG-*GAL4*, females (p value=0.0028, median survival=31 for control, 34 for C9 flies) (C) and males (p value=0.9120, median survival=28.5 for control, 26 for C9 flies) (D), (E-F) with EGN-*GAL4*, females (p value=0.4042, median survival=31 for control, 31 for C9 flies) (E) and males (p value=0.0047, median survival=28.5 for control, 22 for C9 flies) (F), (G-H) with EGT-*GAL4*, females (p value=0.7632, median survival=31 for control, 31 for C9 flies) (G) and males (p value=0.0742, median survival=28.5 for control, 24 for C9 flies) (H), (I-J) with PNG-*GAL4*, females (p value=0.0007, median survival=31 for control, 24.5 for C9 flies) (I) and males (p value<0.0001, median survival=28.5 for control, 14 for C9 flies) (J), (K-L) with SPG-*GAL4*, females (p value<0.0001, median survival=31 for control, 21 for C9 flies) (K) and males (p value<0.0001, median survival=28.5 for control, 12 for C9 flies) (L). The controls in this graph were fixed with subtypes of glial expressions in male and female respectively. Genotypes are labeled above the graph by indicated color. Rearing temperature, age, and sex of animals are labeled within the graph.

**Fig. SD13.** Larval CNS immunostaining and quantification with GR100 expression in surface glia. (A) Third instar larval CNS expressing *UAS-tdTomato* with *PNG-GAL4* only (top panels) or with *UAS-GR100* were immunostained with anti-DsRed (yellow) and anti-HRP (blue, neuronal staining) antibodies. Scale bars represent 100 μm. (B-D) Percent area quantified from (B) tdTom signal represents PNG region, (C) HRP signal represents neurons, and (D) ratio of glia/neuron from the data collected (B) and (C). (E) Third instar larval CNS expressing *UAS-tdTomato* with *SPG-GAL4* only (top panels) or with *UAS-GR100* were immunostained with anti-DsRed (yellow) and anti-HRP (blue, neuronal staining) antibodies. Scale bars represent 100 μm. (F-H) Percent area quantified from (F) tdTom signal represents PNG region, (G) HRP signal represents neurons, and (H) ratio of glia/neuron from the data collected (F) and (G). See **METHODS** and previous report ^6,7^ for detailed quantification methods.

**Fig. SD14.** Larval CNS immunostaining and quantification with flies expressing GR100 in astrocyte-like glia. (A) Third instar larval CNS expressing *UAS-tdTomato* with *ALG-GAL4* only (top panels) or with *UAS-GR100* were immunostained with anti-DsRed (yellow) and anti-HRP (blue, neuronal staining) antibodies. Scale bars represent 100 μm. (B-D) Percent area quantified from (B) tdTom signal represents PNG region, (C) HRP signal represents neurons, and (D) ratio of glia/neuron from the data collected (B) and (C).

**Fig. SD15.** Climbing test of non-treated or UDCA-treated flies expressing GR100 variant by nSyb, PNG and SPG*-GAL4* drivers. (A-B) GR100 expressing by nSyb -*GAL4* driver flies, 5-day-old females (A) and males (B). (C-F) GR100 expressing by SPG-*GAL4* driver flies, 5-day-old females (C) and males (D), 20-day-old females (E) and males (F). (G-J) GR100 expressing by PNG-*GAL4* driver flies, 5-day-old females (G) and males (H), 20-day-old females (I) and males (J). Box-and-whisker plot represent the percent of flies crossed midline. All experiments were performed five times after sufficient recovering period. Genotypes are labeled below the graph. Rearing temperature, age, and sex of animals are labeled within the graph.

**Fig. SD16.** Lifespan assay of non-treated or UDCA-treated flies expressing GR100 variant by nSyb, PNG and SPG*-GAL4* drivers. (A and B) GR100 female (p value<0.0001, median survival=12 for control, 10 for treated flies) and male (p value=0.3776, median survival=5 for control, 6 for treated flies) by *nSyb-GAL4* driver. (C and D) GR100 female (p value<0.0001, median survival=55 for control, 26 for treated flies) and male (p value<0.0001, median survival=49 for control, 16 for treated flies) by *SPG-GAL4* driver. (E and F) GR100 female (p value=0.0833, median survival=30 for control, 24 for treated flies) and male (p value=0.0002, median survival=19 for control, 14 for treated flies) by *PNG-GAL4* driver.
