# Supplementary figures and images for: Surface glia for modeling ALS-FTD-associated mutant C9orf72 toxicity in the nervous system of Drosophila
Source: Genes Dis. 2025 Apr 5;13(2):101629. doi: 10.1016/j.gendis.2025.101629 (PMC12596593; doi:10.1016/j.gendis.2025.101629)

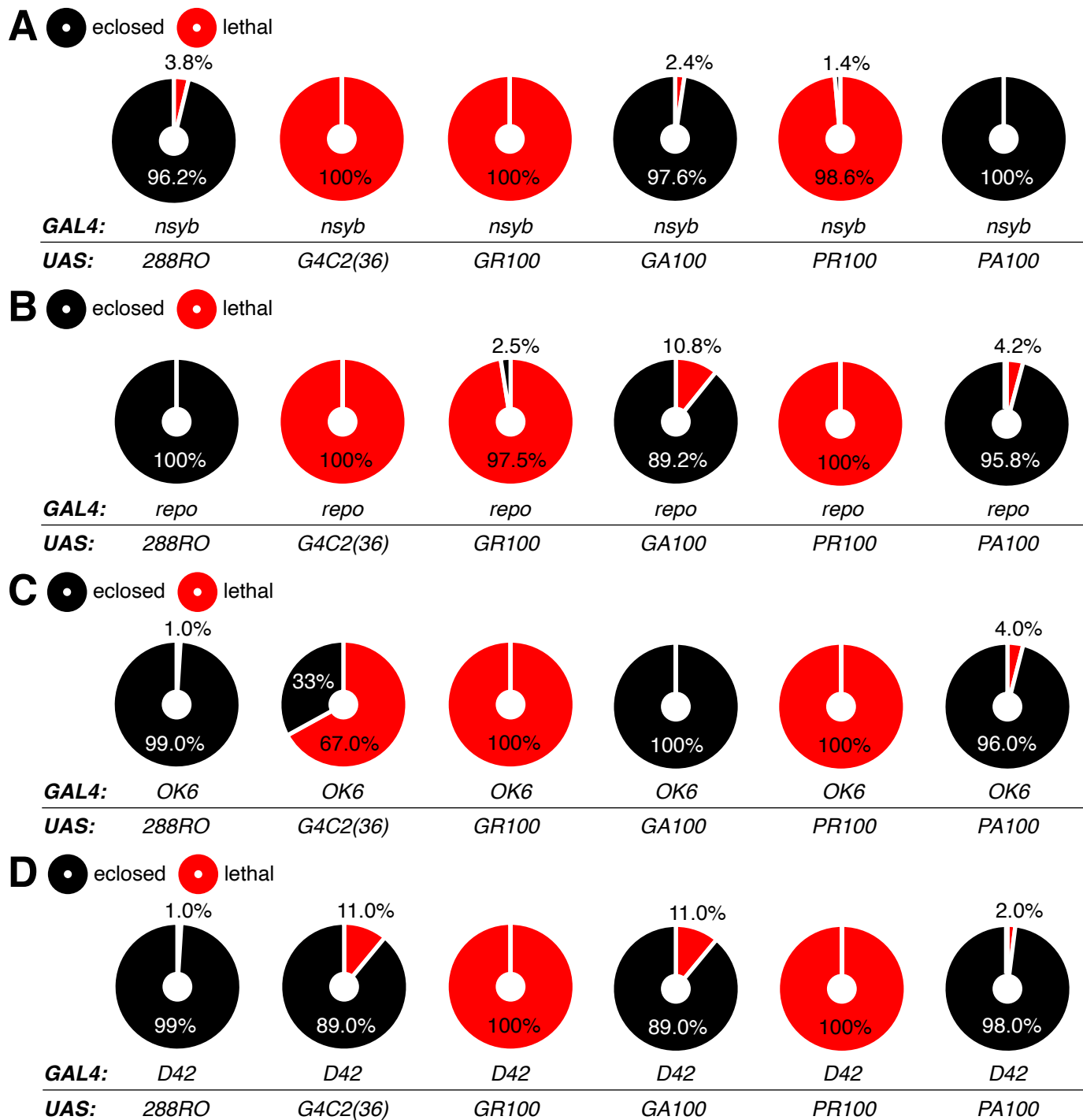

**Wei., SD.1**

Supplement: Multimedia component 2 [file mmc2.pdf]

**A** ● *UAS/TM3* ● *UAS/GAL4*

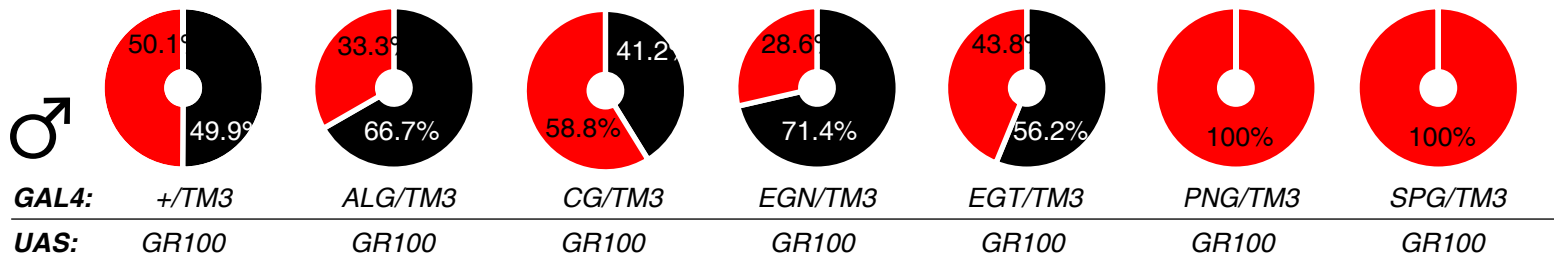

*Wei., SD.2*

Supplement: Multimedia component 3 [file mmc3.pdf]

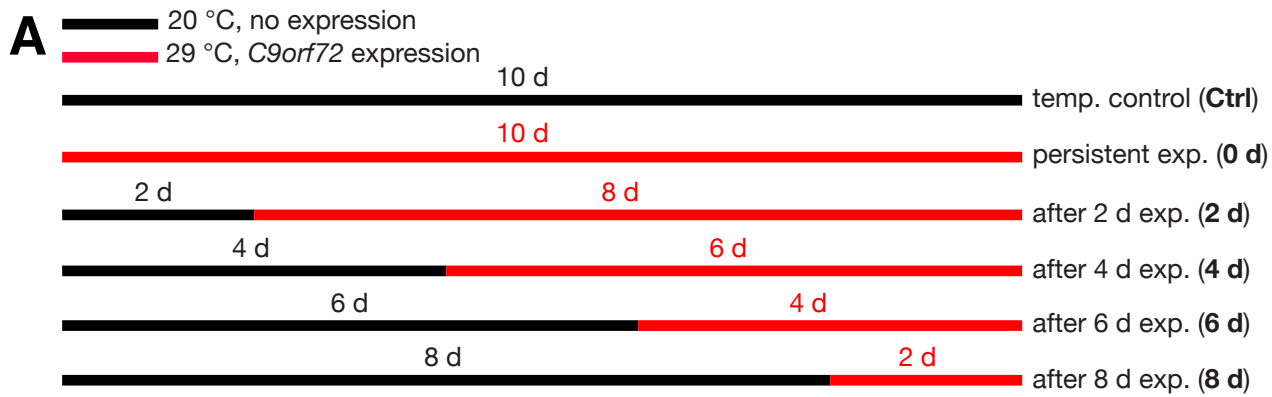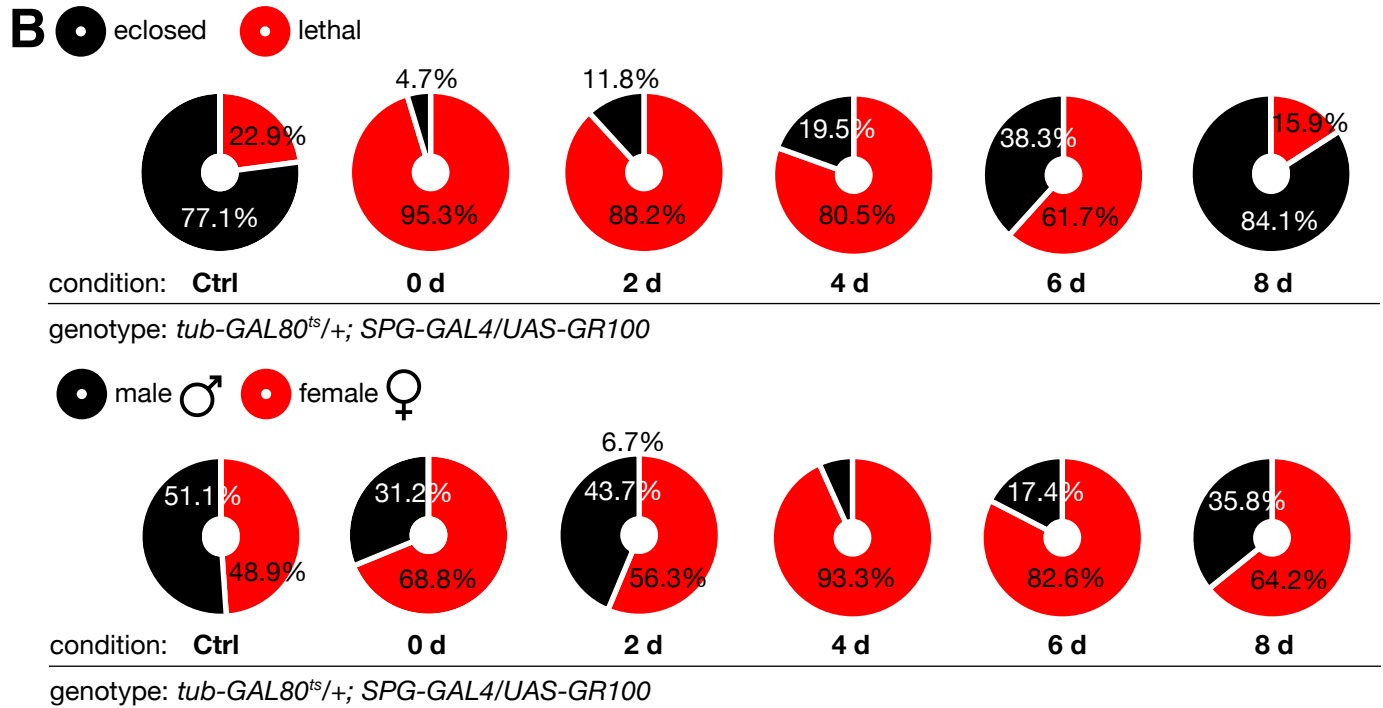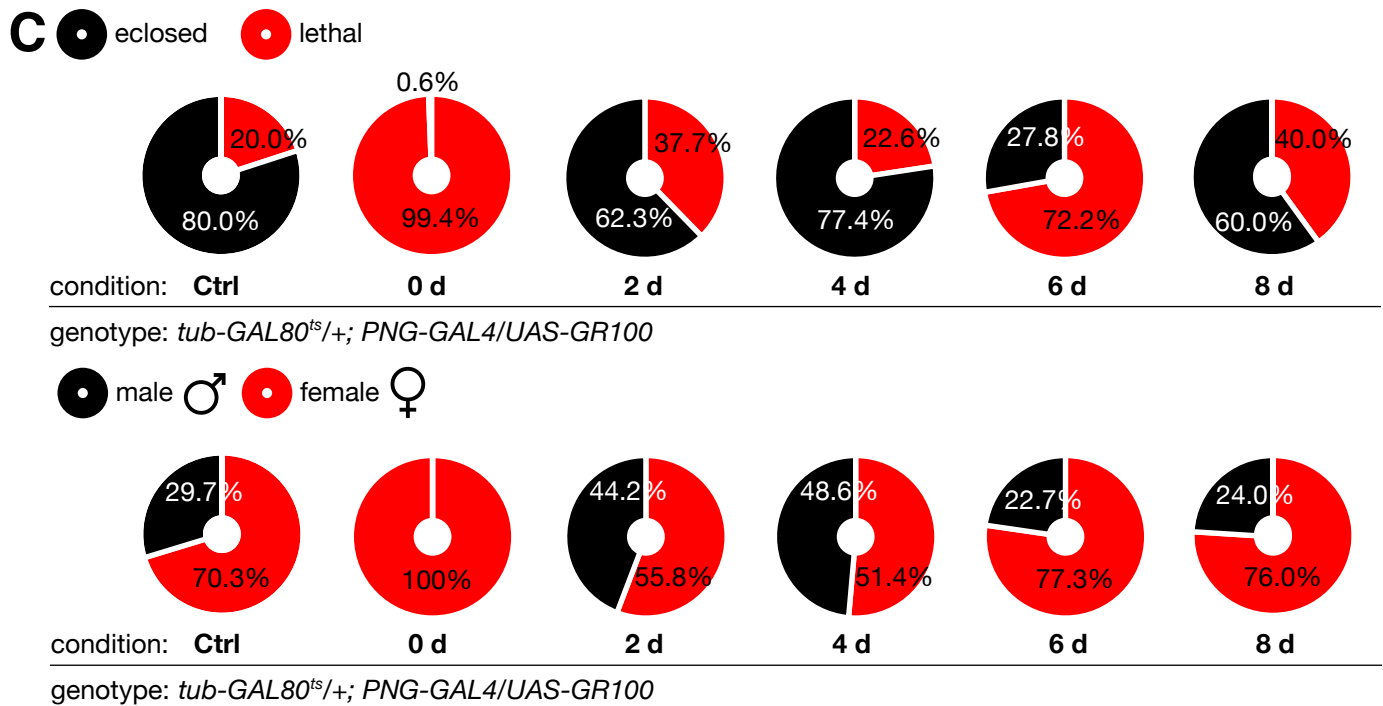

Supplement: Multimedia component 4 [file mmc4.pdf]

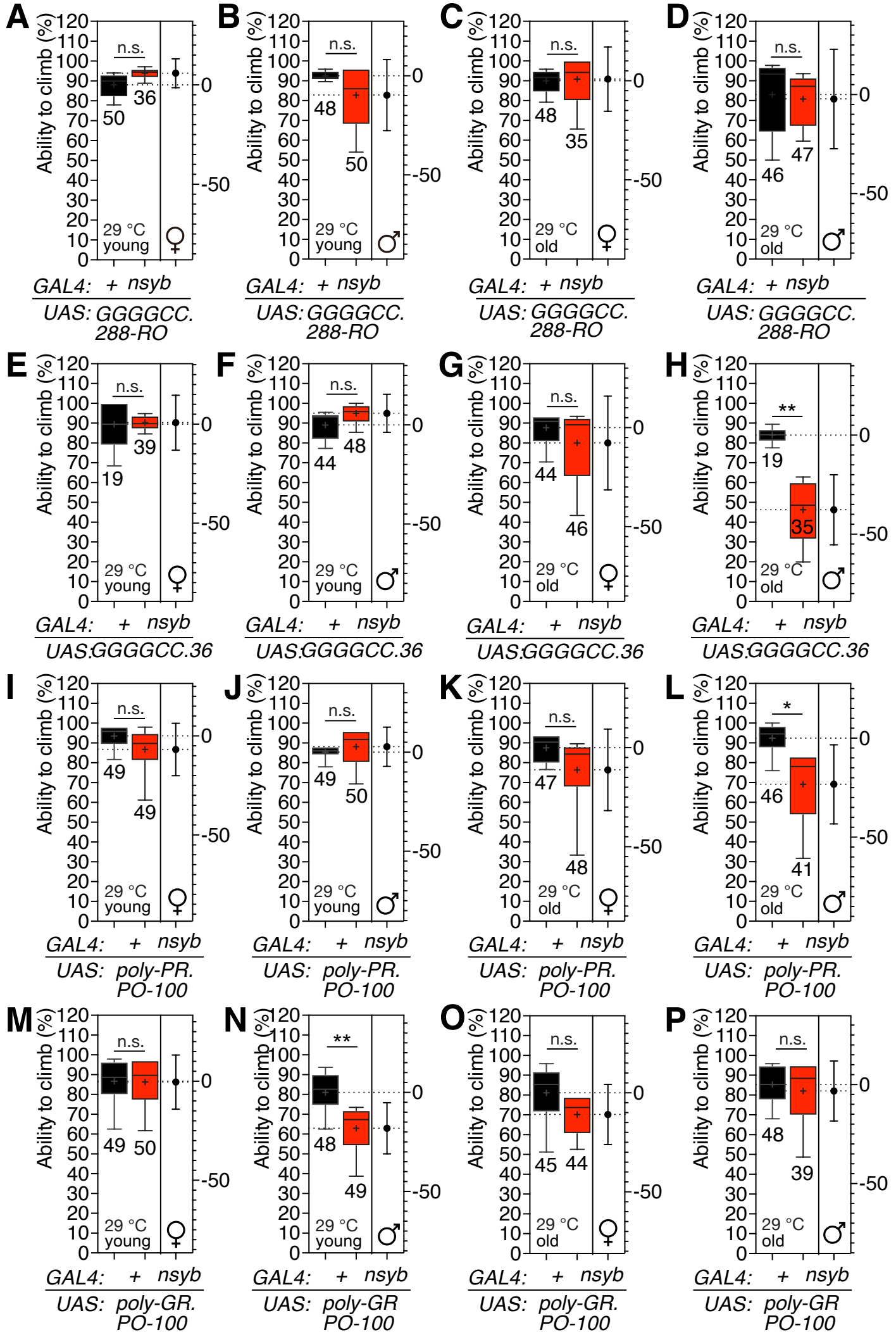

Supplement: Multimedia component 5 [file mmc5.pdf]

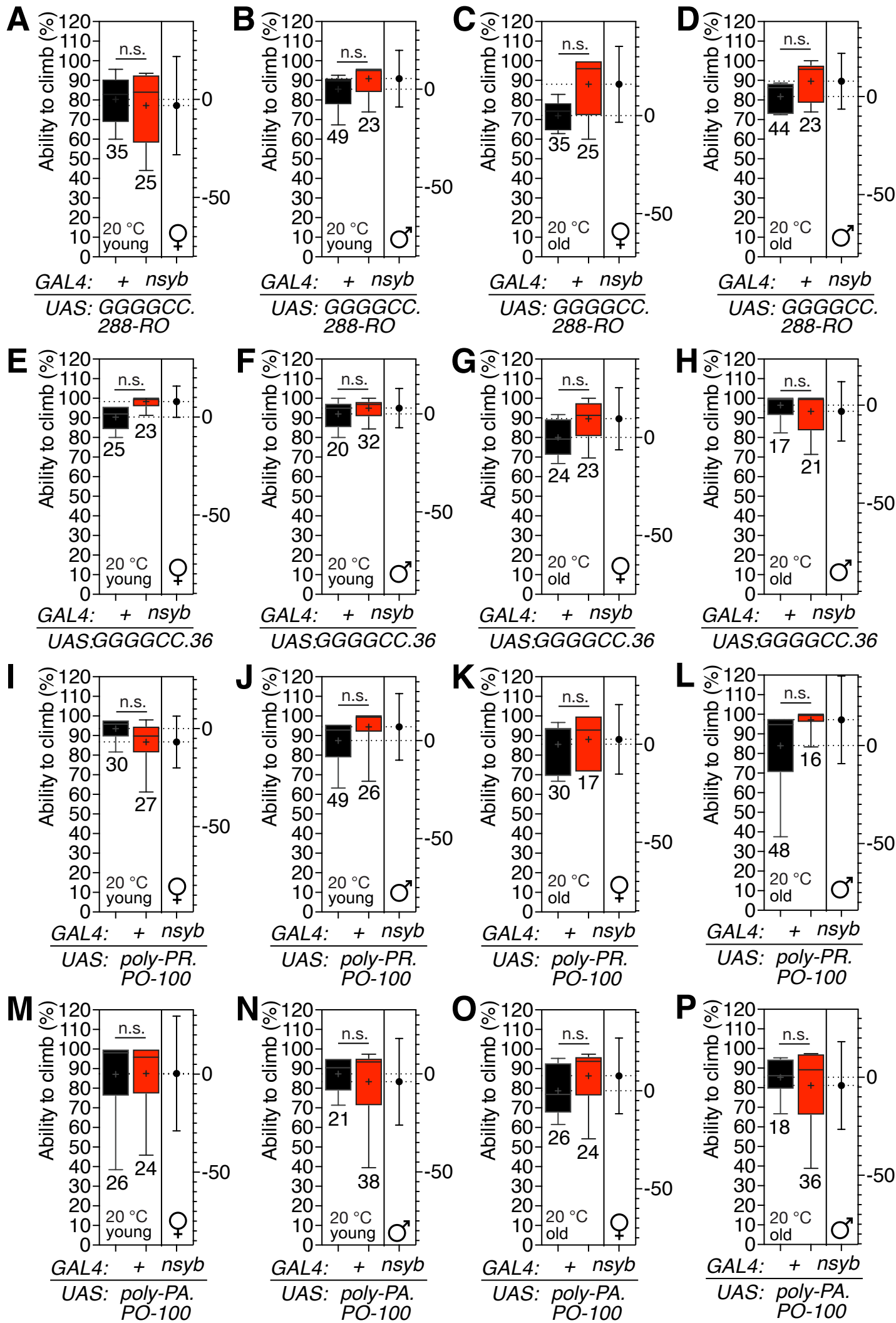

Supplement: Multimedia component 6 [file mmc6.pdf]

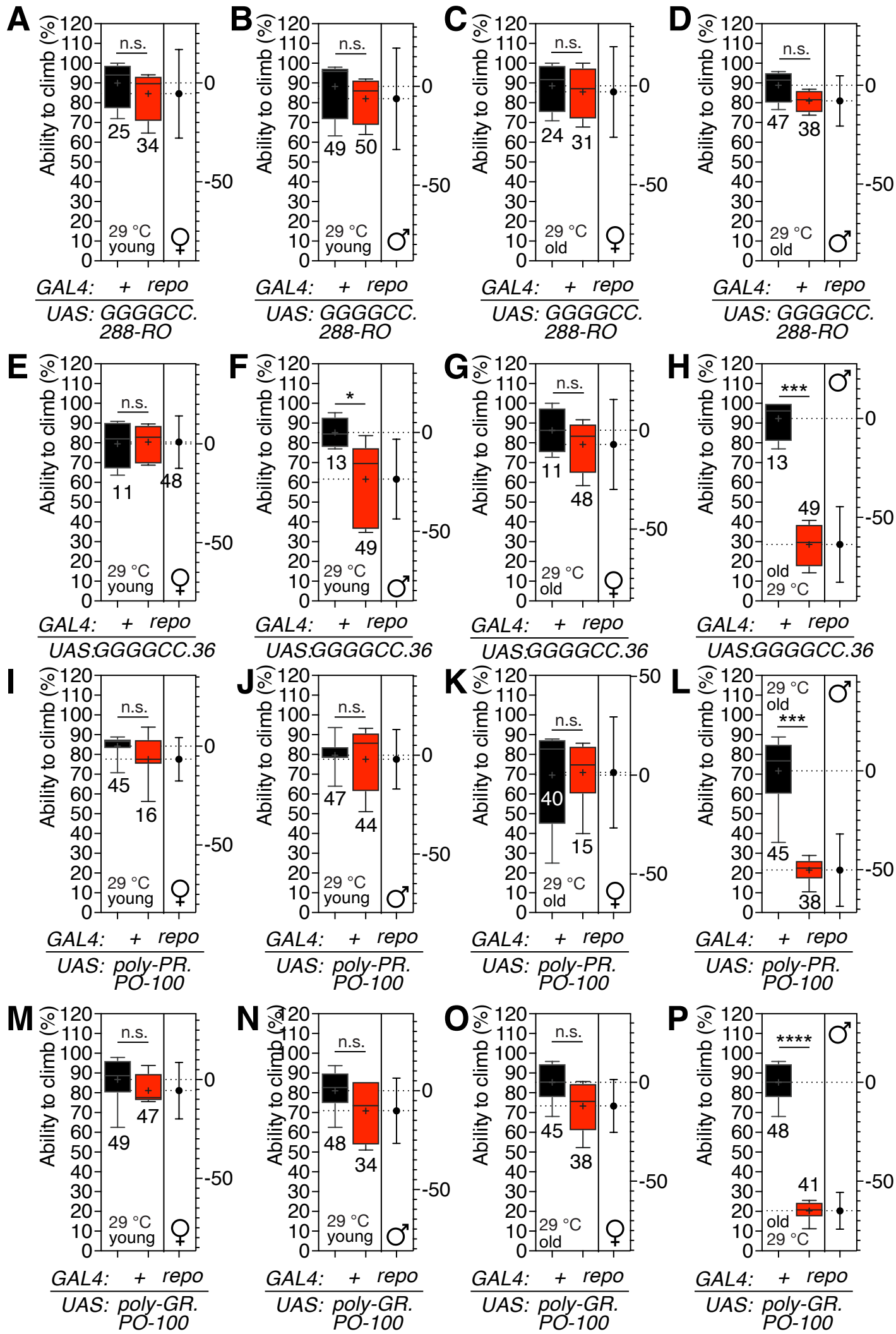

Supplement: Multimedia component 7 [file mmc7.pdf]

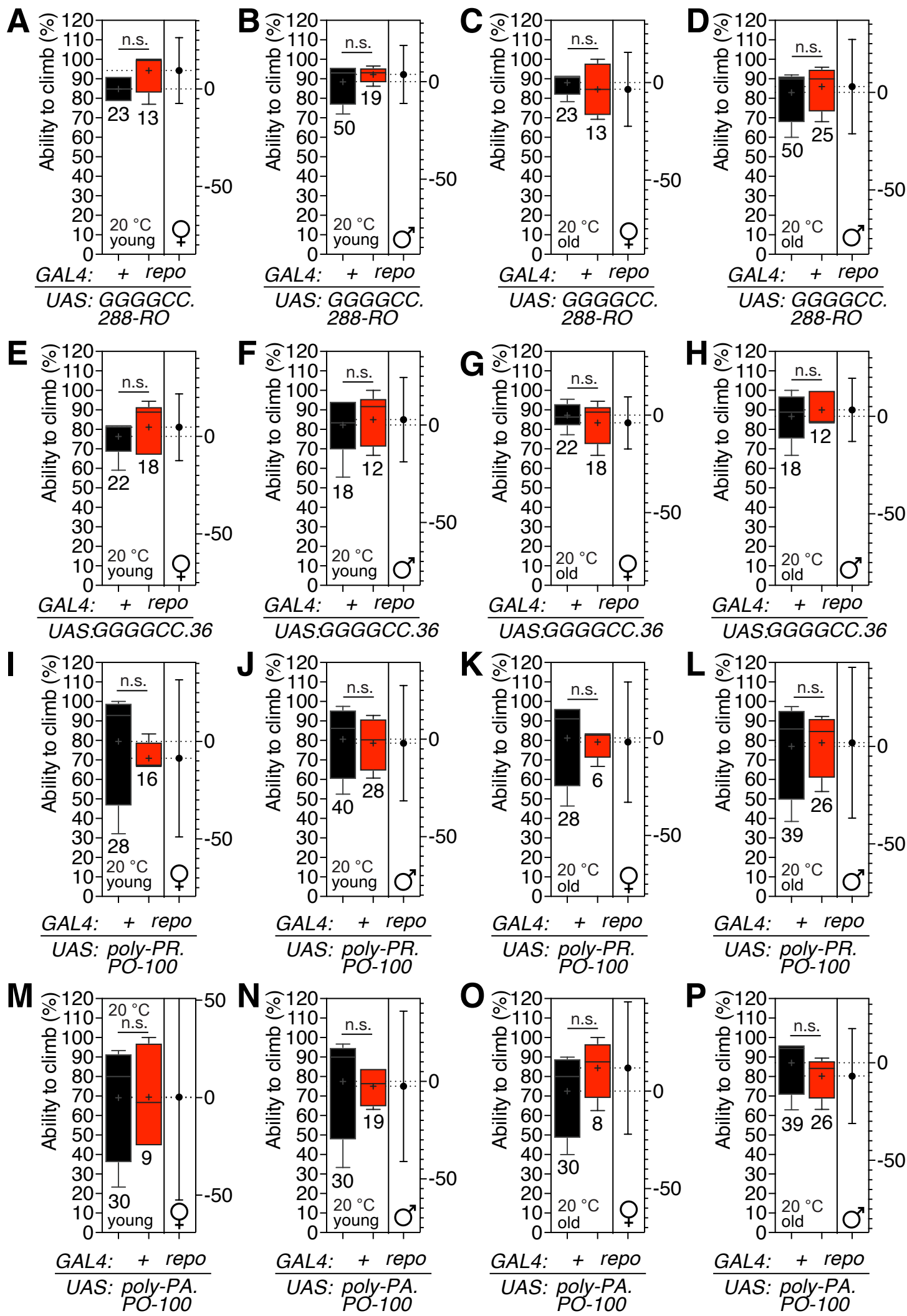

Supplement: Multimedia component 8 [file mmc8.pdf]

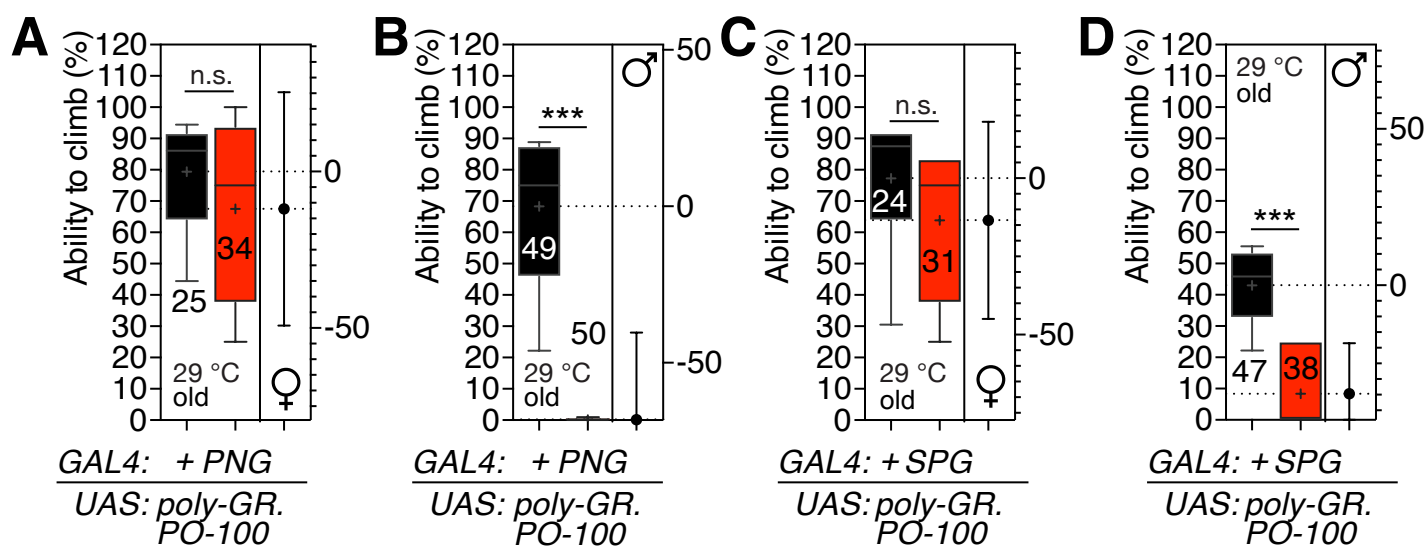

**Wei., SD.8**

Supplement: Multimedia component 9 [file mmc9.pdf]

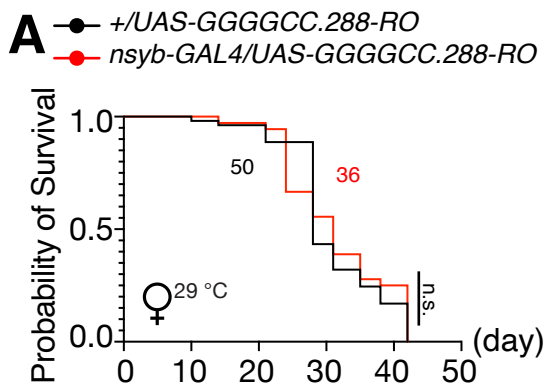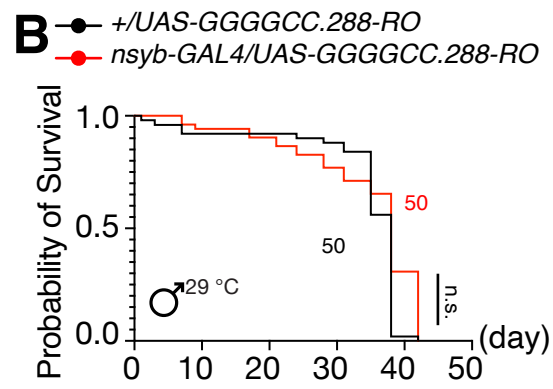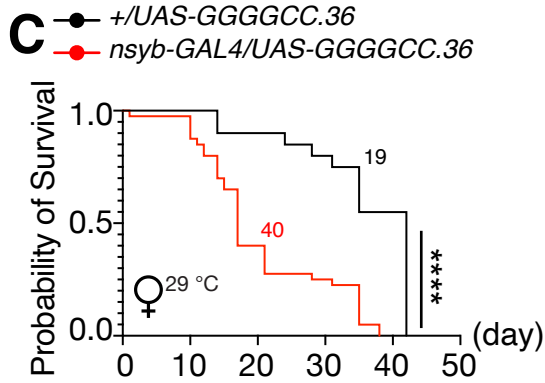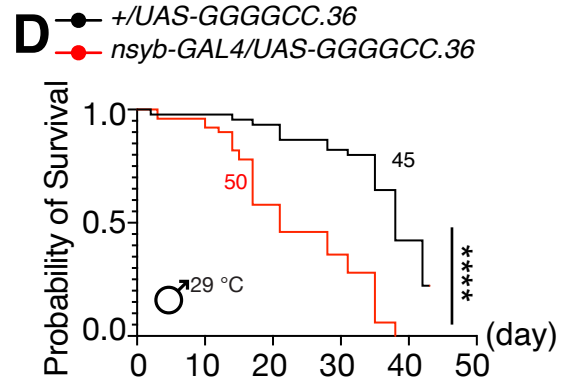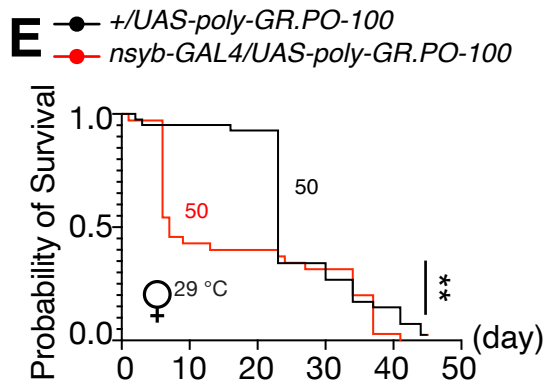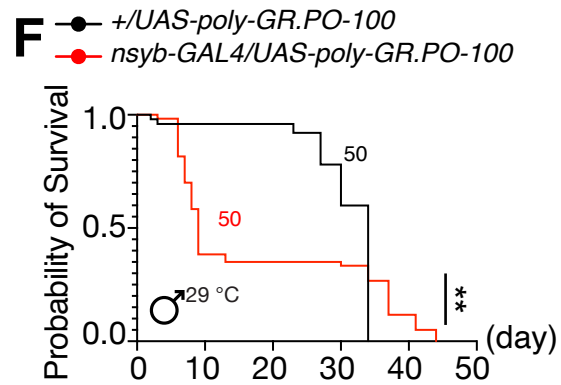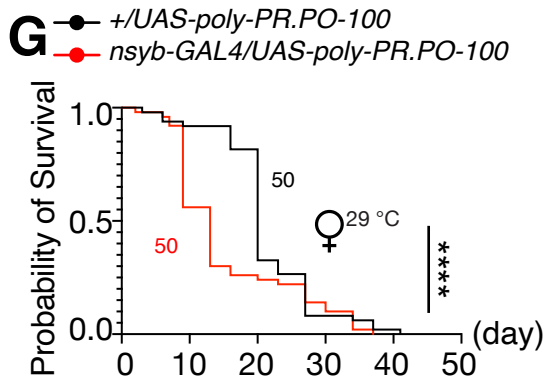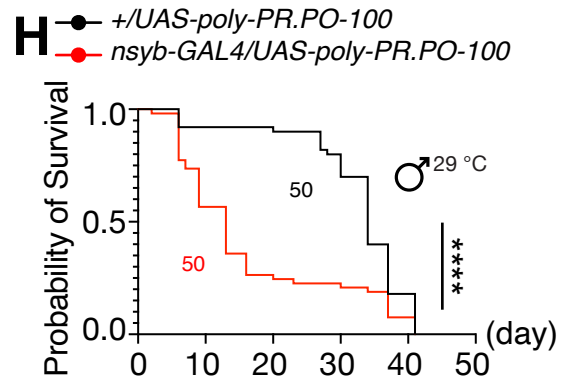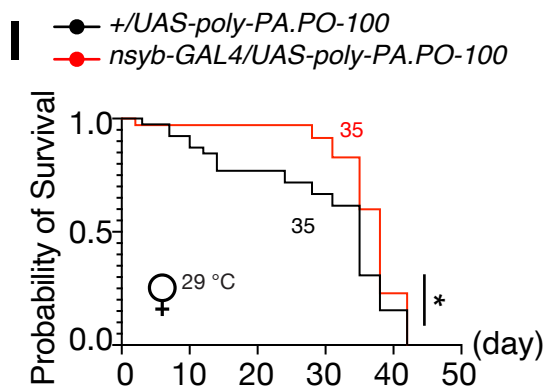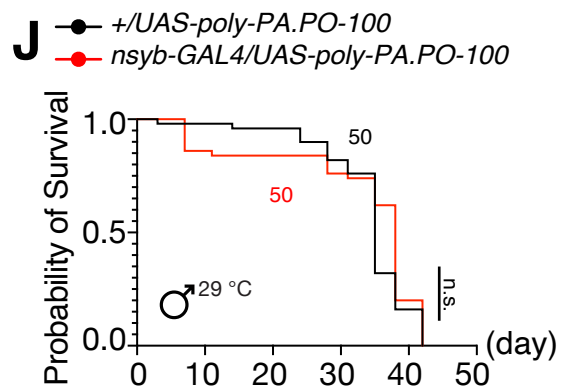

Supplement: Multimedia component 10 [file mmc10.pdf]

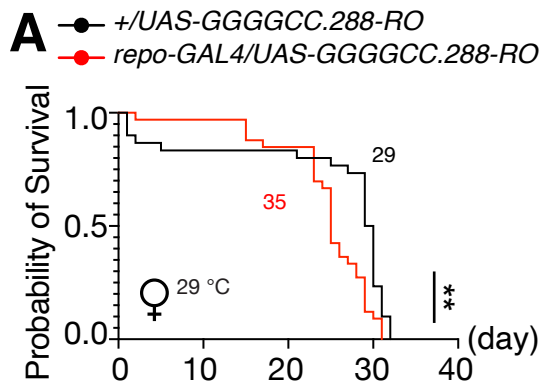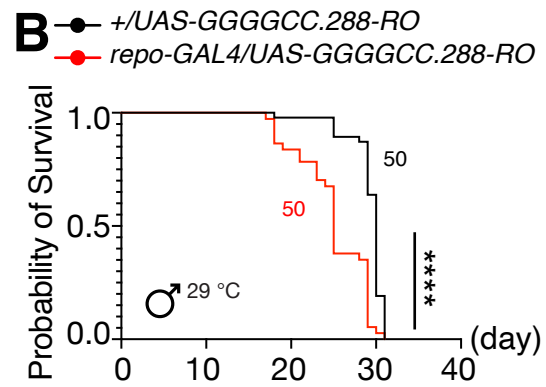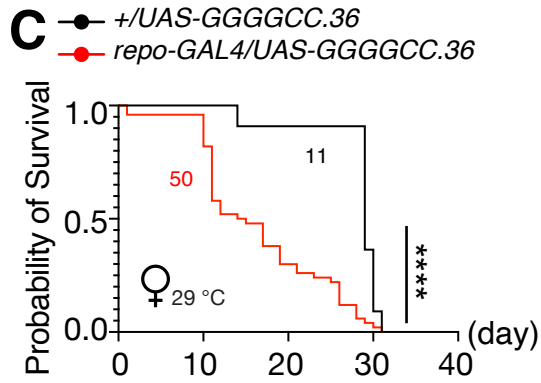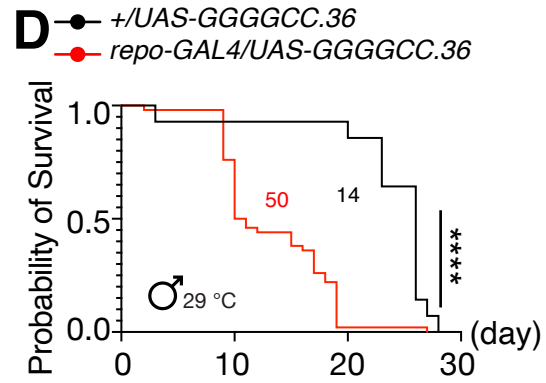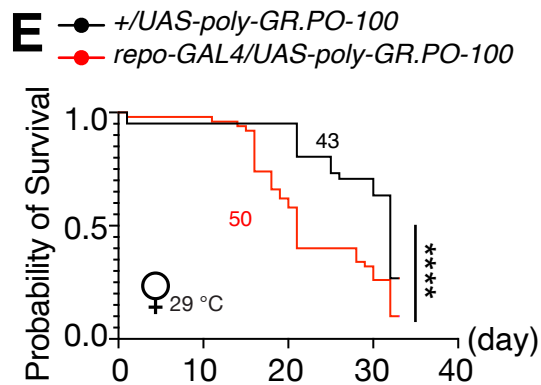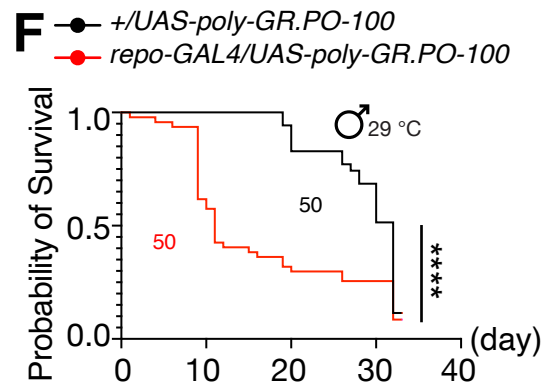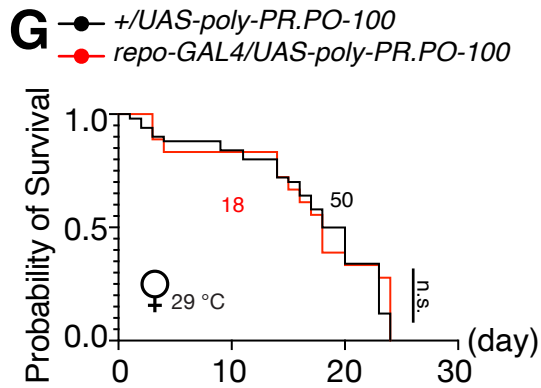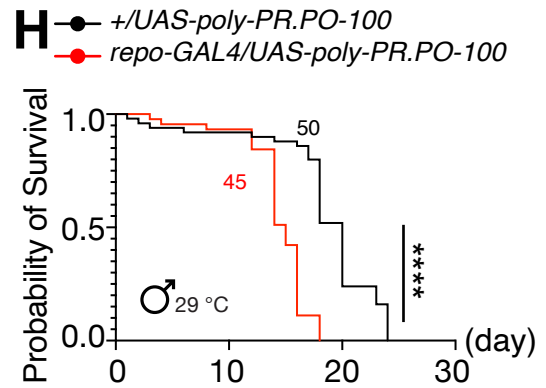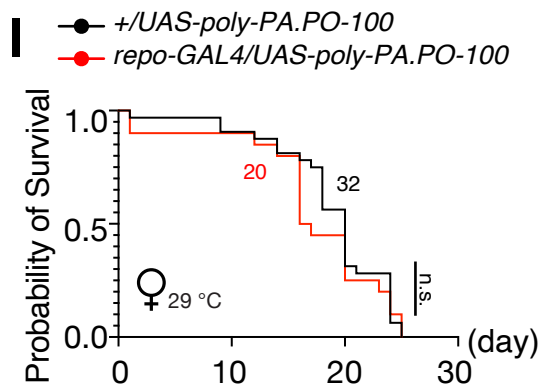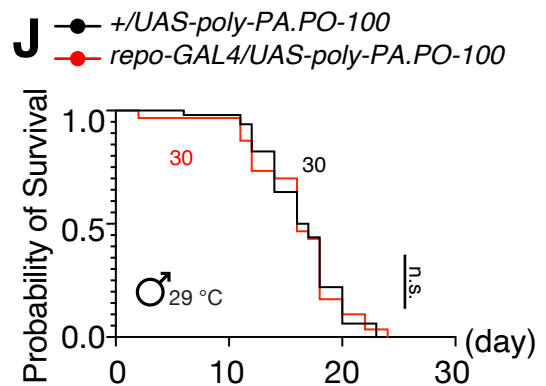

Supplement: Multimedia component 11 [file mmc11.pdf]

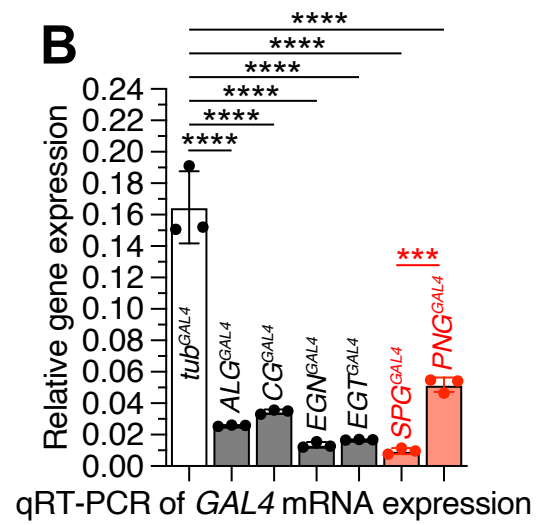

***Wei., SD.11***

Supplement: Multimedia component 12 [file mmc12.pdf]

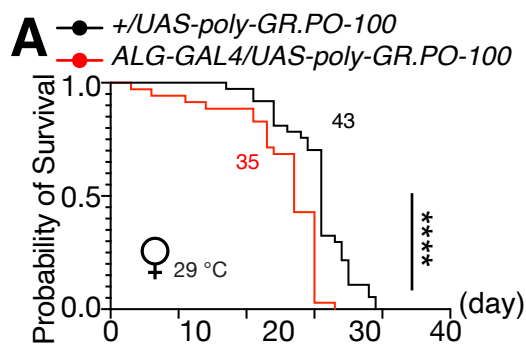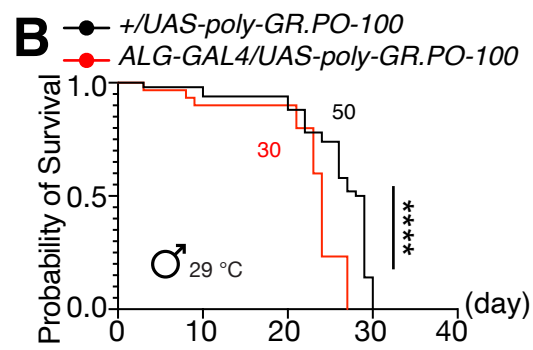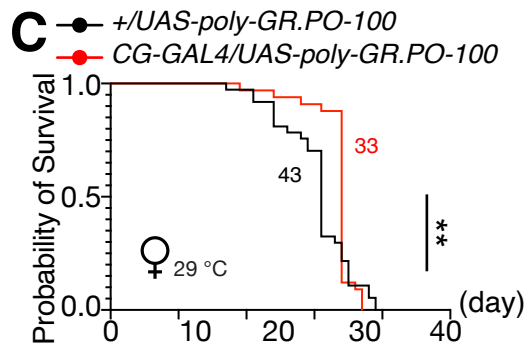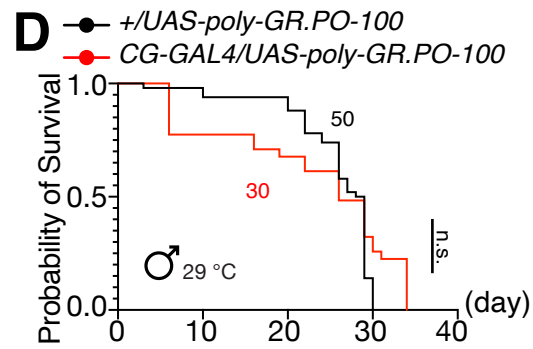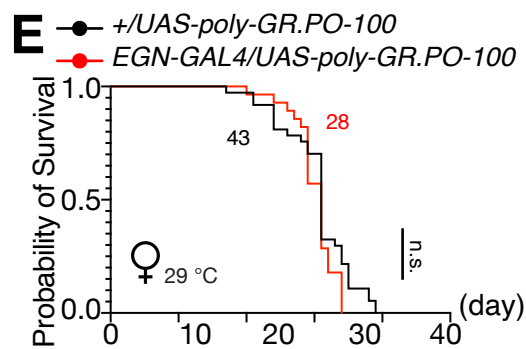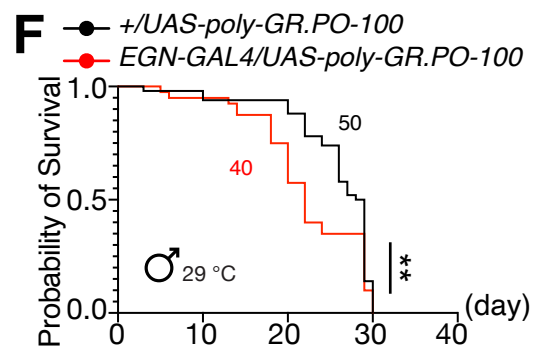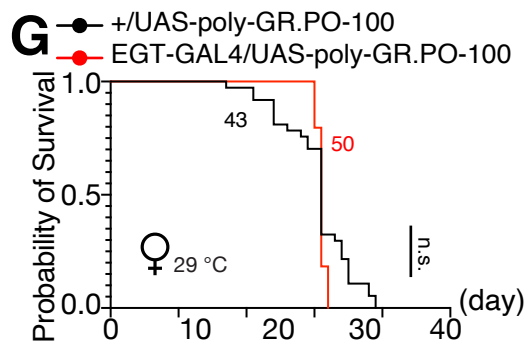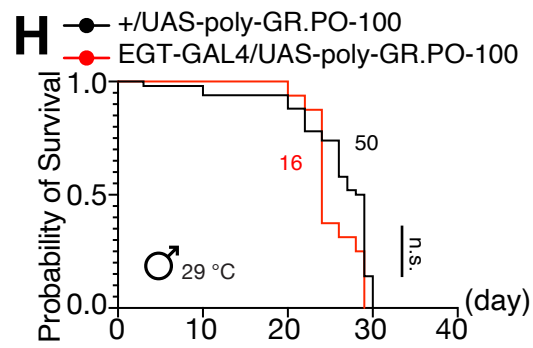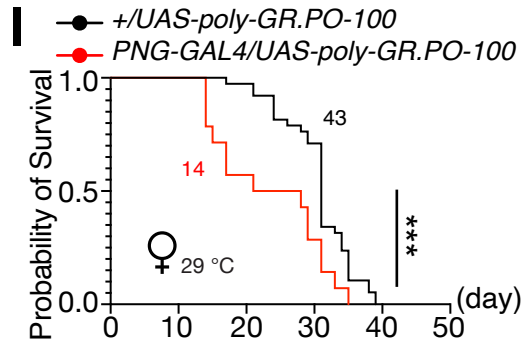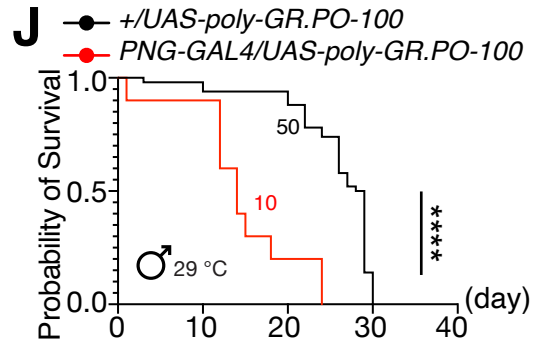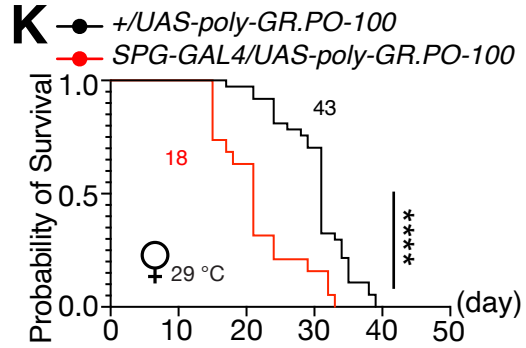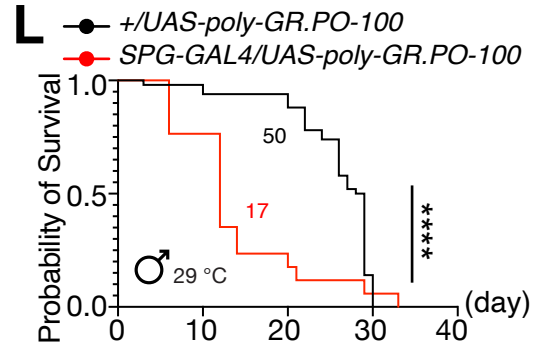

Supplement: Multimedia component 13 [file mmc13.pdf]

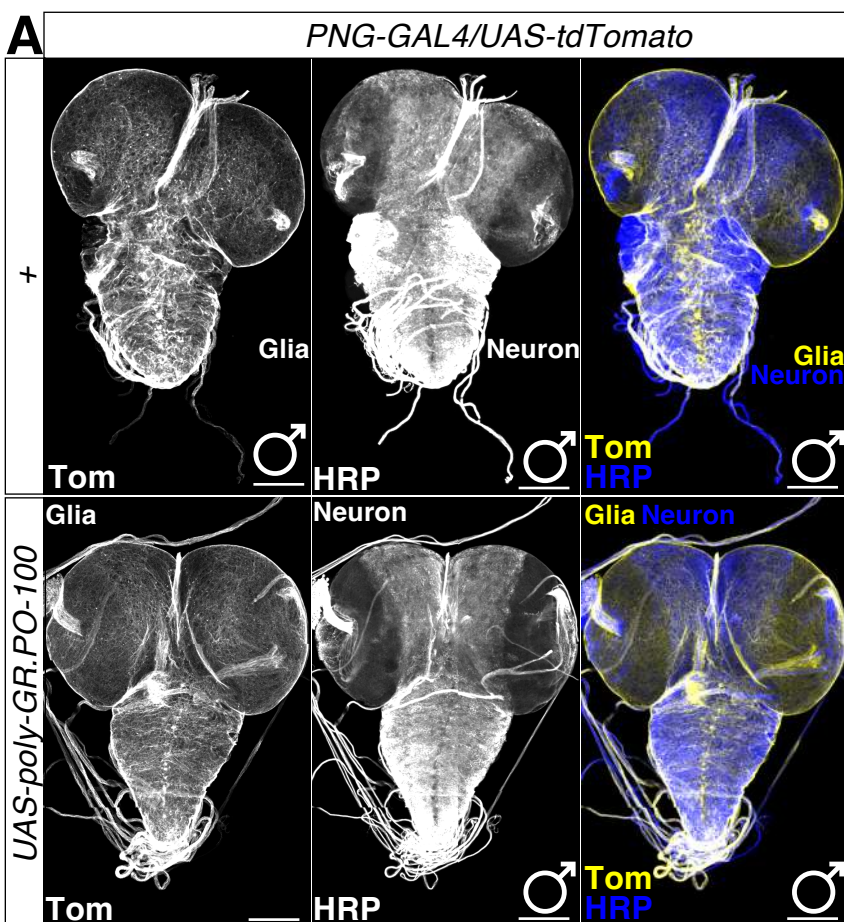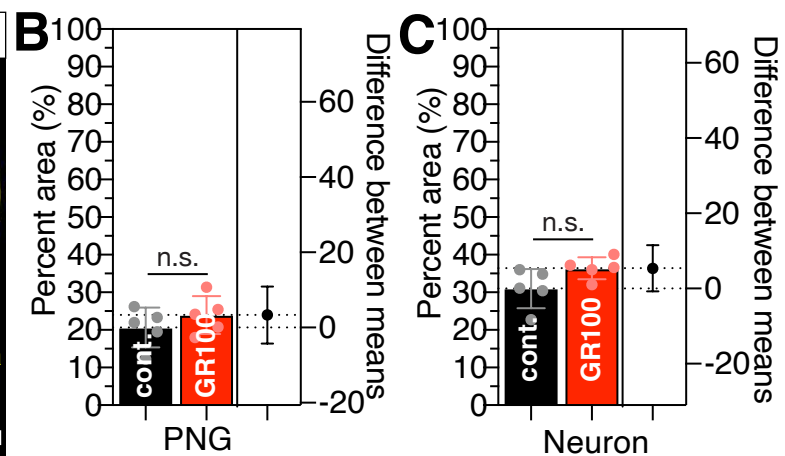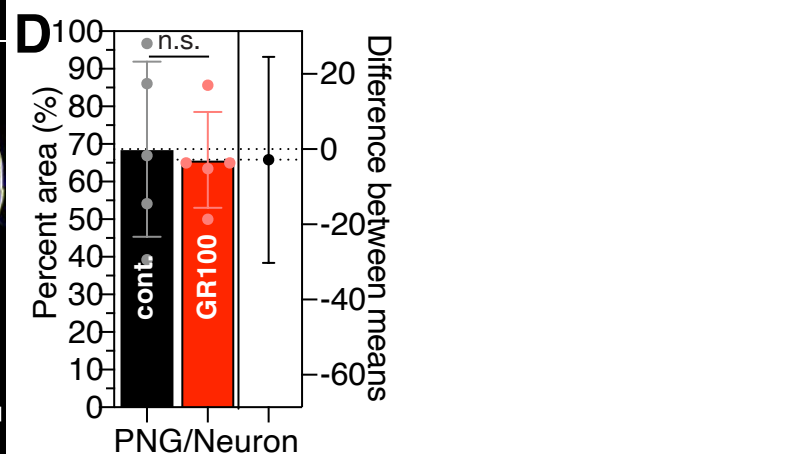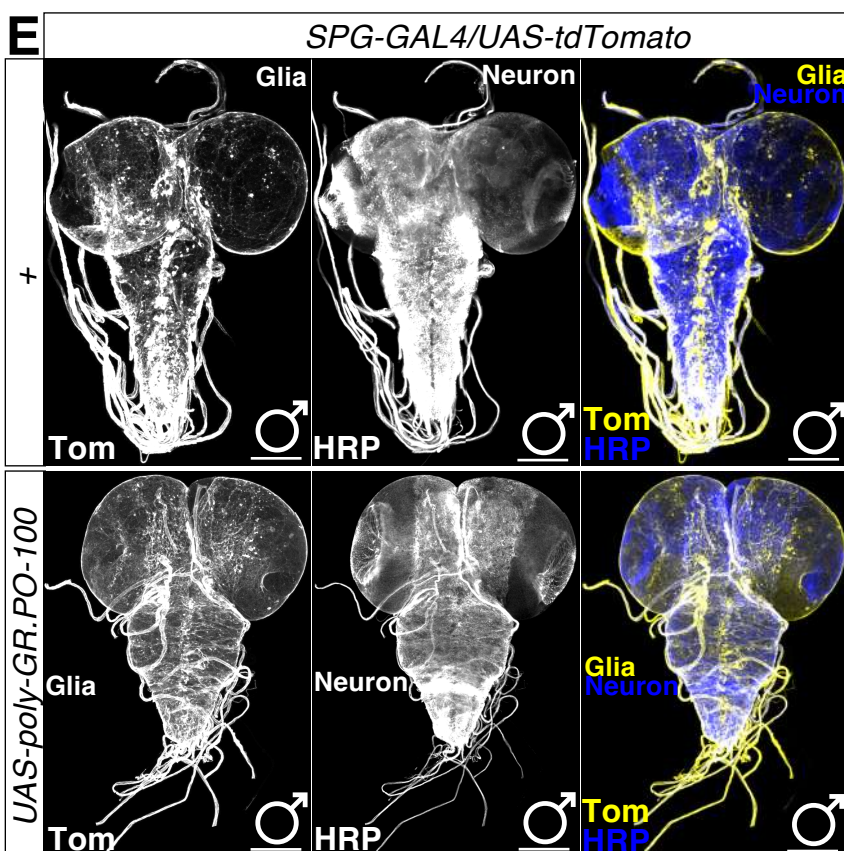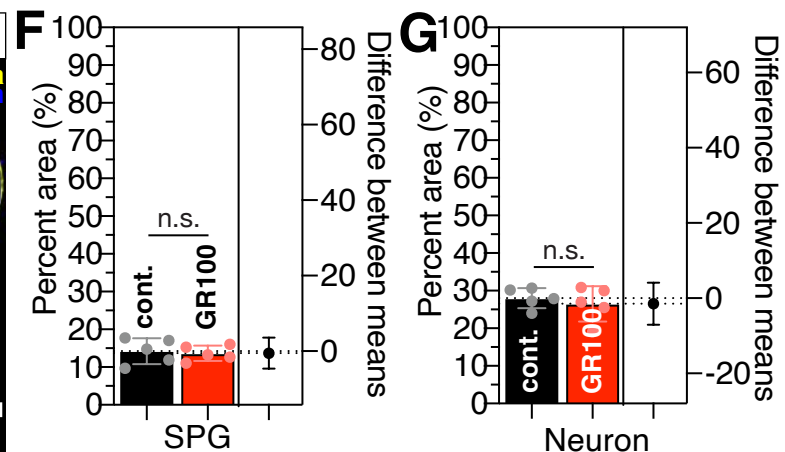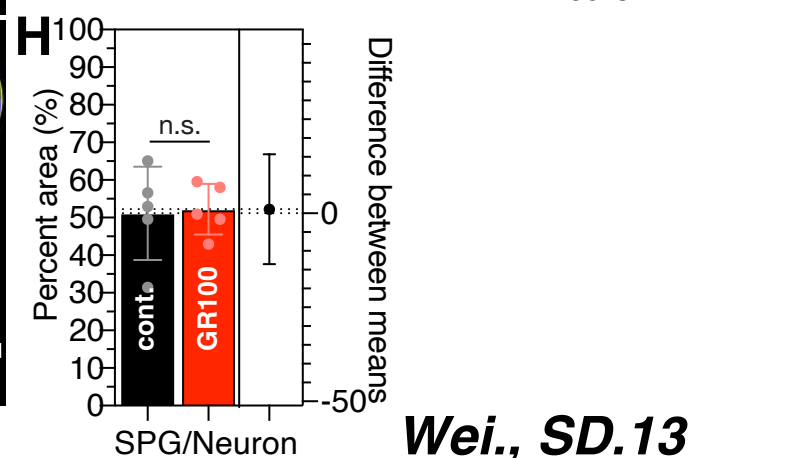

**Wei., SD.13**

Supplement: Multimedia component 14 [file mmc14.pdf]

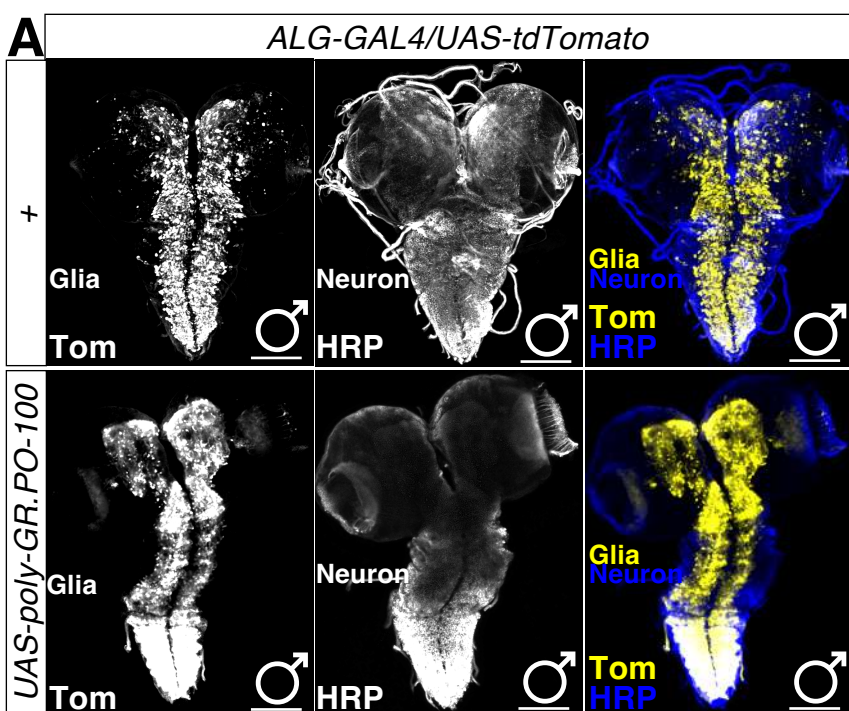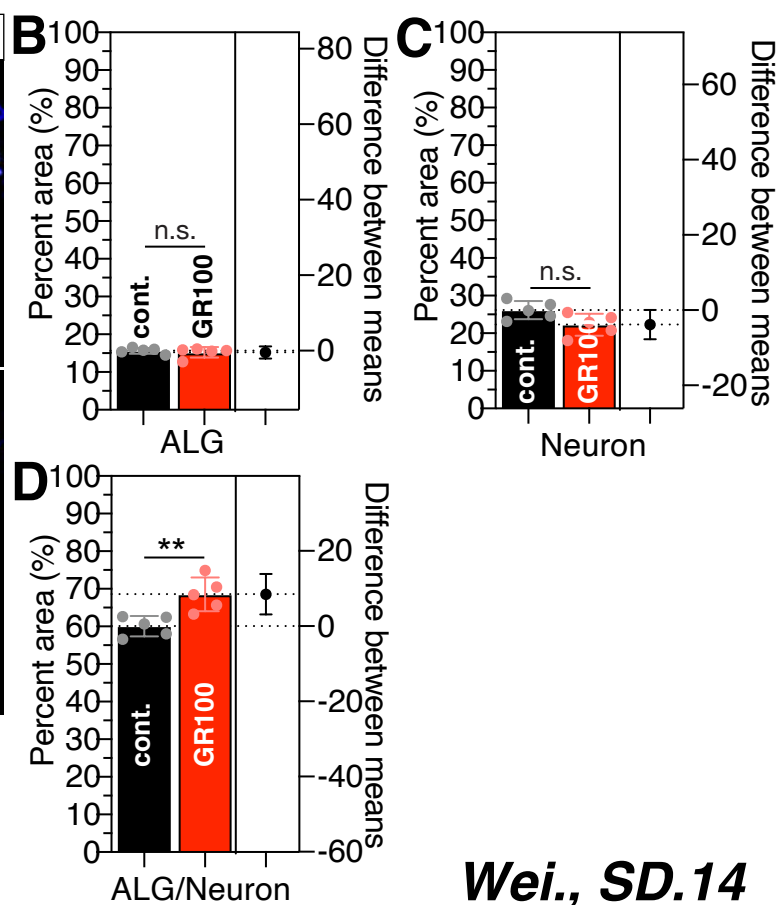

**Wei., SD.14**

Supplement: Multimedia component 15 [file mmc15.pdf]

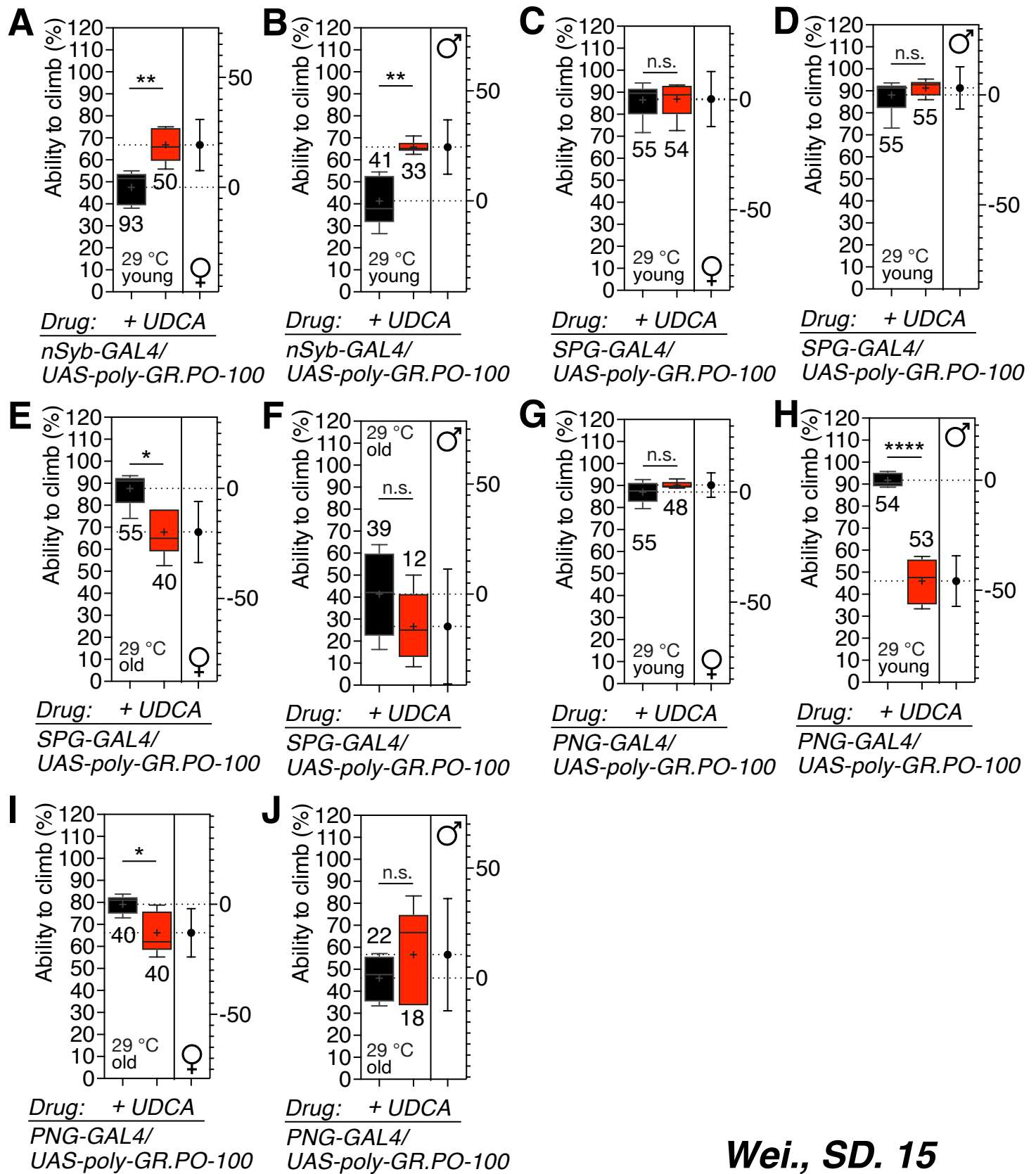

Supplement: Multimedia component 16 [file mmc16.pdf]

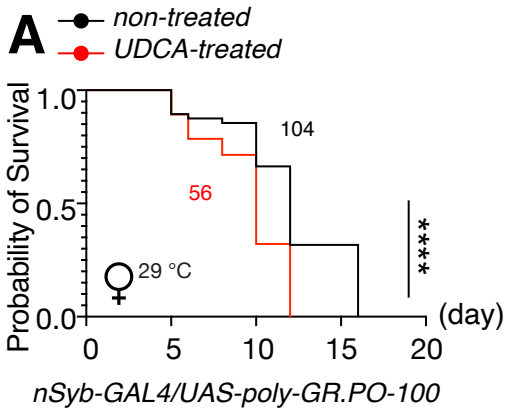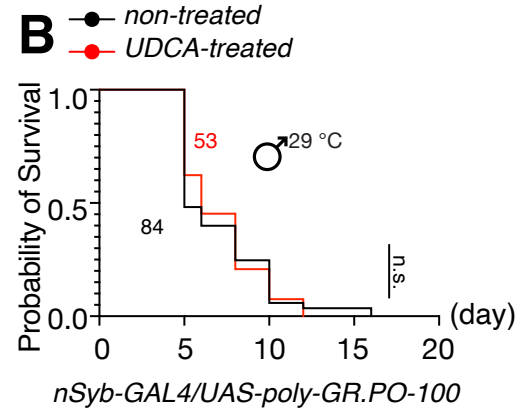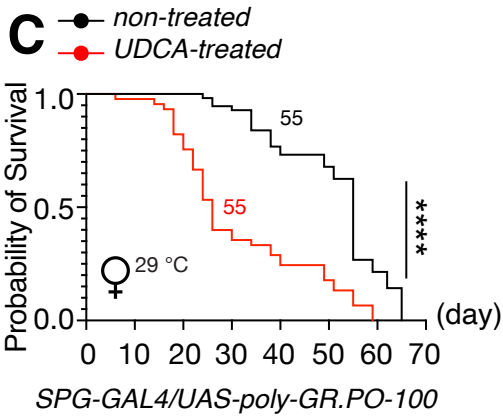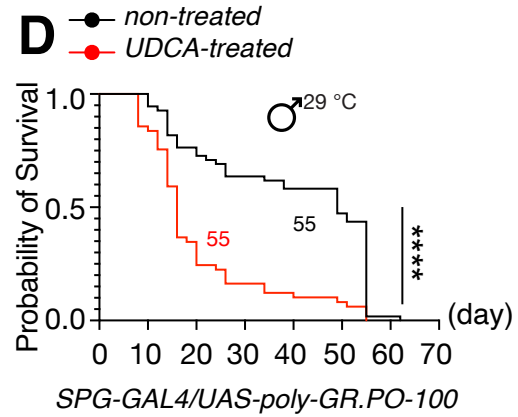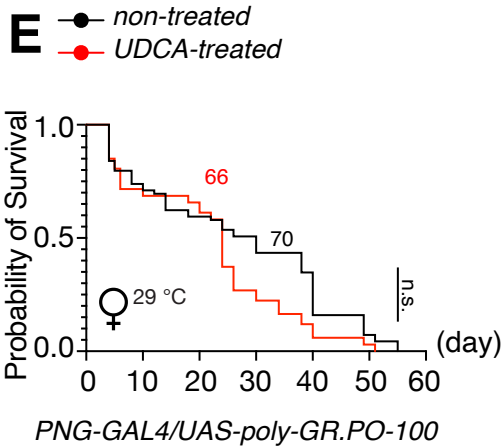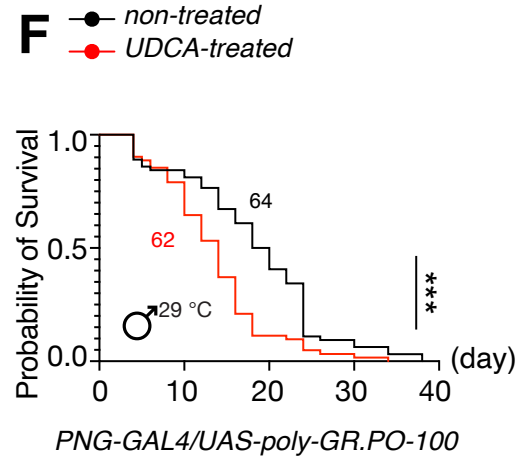

**Wei., SD. 16**

Supplement: Multimedia component 17 [file mmc17.pdf]
